# Whole Genome Analyses of Chinese Population and *De Novo* Assembly of A Northern Han Genome

**DOI:** 10.1016/j.gpb.2019.07.002

**Published:** 2019-09-05

**Authors:** Zhenglin Du, Liang Ma, Hongzhu Qu, Wei Chen, Bing Zhang, Xi Lu, Weibo Zhai, Xin Sheng, Yongqiao Sun, Wenjie Li, Meng Lei, Qiuhui Qi, Na Yuan, Shuo Shi, Jingyao Zeng, Jinyue Wang, Yadong Yang, Qi Liu, Yaqiang Hong, Lili Dong, Zhewen Zhang, Dong Zou, Yanqing Wang, Shuhui Song, Fan Liu, Xiangdong Fang, Hua Chen, Xin Liu, Jingfa Xiao, Changqing Zeng

**Affiliations:** 1 Beijing Institute of Genomics, Chinese Academy of Sciences, Beijing 100101, China; 2 BIG Data Center, Beijing Institute of Genomics, Chinese Academy of Sciences, Beijing 100101, China; 3 CAS Key Laboratory of Genomic and Precision Medicine, Beijing Institute of Genomics, Chinese Academy of Sciences, Beijing 100101, China; 4 CAS Key Laboratory of Genome Sciences and Information, Beijing Institute of Genomics, Chinese Academy of Sciences, Beijing 100101, China; 5 University of Chinese Academy of Sciences, Beijing 100049, China

**Keywords:** *De novo* assembly, Reference genome, Variation map, Phenotype association, Large population

## Abstract

To unravel the genetic mechanisms of disease and physiological traits, it requires comprehensive sequencing analysis of large sample size in Chinese populations. Here, we report the primary results of the Chinese Academy of Sciences Precision Medicine Initiative (CASPMI) project launched by the Chinese Academy of Sciences, including the ***de novo* assembly** of a northern Han **reference genome** (NH1.0) and whole genome analyses of 597 healthy people coming from most areas in China. Given the two existing reference genomes for Han Chinese (YH and HX1) were both from the south, we constructed NH1.0, a new reference genome from a northern individual, by combining the sequencing strategies of PacBio, 10× Genomics, and Bionano mapping. Using this integrated approach, we obtained an N50 scaffold size of 46.63 Mb for the NH1.0 genome and performed a comparative genome analysis of NH1.0 with YH and HX1. In order to generate a genomic **variation map** of Chinese populations, we performed the whole-genome sequencing of 597 participants and identified 24.85 million (M) single nucleotide variants (SNVs), 3.85 M small indels, and 106,382 structural variations. In the association analysis with collected phenotypes, we found that the T allele of rs1549293 in *KAT8* significantly correlated with the waist circumference in northern Han males. Moreover, significant genetic diversity in *MTHFR*, *TCN2*, *FADS1*, and *FADS2*, which associate with circulating folate, vitamin B12, or lipid metabolism, was observed between northerners and southerners. Especially, for the homocysteine-increasing allele of rs1801133 (*MTHFR* 677T), we hypothesize that there exists a “comfort” zone for a high frequency of 677T between latitudes of 35–45 degree North. Taken together, our results provide a high-quality northern Han reference genome and novel population-specific data sets of genetic variants for use in the personalized and precision medicine.

## Introduction

To understand the genetic basis of disease and develop individualized medication, the Human Genome Project generated the first human reference genome, which was based on the Caucasian genetic background [1], [2], [3]. Despite the consistent updating of the reference genome to the latest version GRCh38, it is still highly demanded to construct the regional reference genomes of various ethnic groups for the advanced medical and population studies. Since the release of the first Asian individual genome (the YH genome from southern China) over a decade ago [2], recent advances in both experimental and *in silico* technologies have enabled the *de novo* assembly of individual human genomes with remarkably improved completeness and accuracy. For instance, the Korean genome AK1 was reported using multiple sequencing methods in 2016 [4], and subsequently an updated *de novo* assembly of the YH genome was generated utilizing a haplotype-based approach [5]. In addition, another Chinese reference genome also from the south, HX1, was constructed using single molecular sequencing and NanoChanel array [6]. However, as evidenced by DNA markers and SNP array analyses, northern and southern Chinese populations have undergone significant genetic differentiation during the prehistoric times of agricultural civilization [7], [8], [9], [10]. Thus, population studies will largely benefit from the creation of a northern Chinese reference genome, which is currently lacking.

Unraveling the individualized genetic mechanisms of disease and physiological traits requires comprehensive sequencing analysis of population samples [11], [12]. Following the initial efforts of the International HapMap Project and the 1000 Genomes Project (1KGP) [13], [14], extensive genome-wide association studies (GWAS) have been conducted globally for over a decade, resulting in over 60 K of SNP-trait correlations being reported in the current GWAS Catalog [15]. Furthermore, taking advantage of continuing breakthroughs in next-generation sequencing, several national whole-genome sequencing (WGS)-based projects have been completed in recent years. For instance, parent-offspring samples were sequenced in the Genome of the Netherlands (GoNL, 250 trio or larger family samples, average 13×) and in the GenomeDenmark (50 trio family samples, average 78×) [16], [17]. A milestone in population studies was the WGS of 2638 Icelanders (≥10 × coverage) plus the array-based genotyping of another 100,000 individuals, which resulted in both the identification of disease-related variants and the discovery of the common lineage for this remarkably homogenous population [18]. Aiming to characterize somatic mutations and rare variants in patients with cancer and rare diseases, the UK10K project includes the WGS (∼7×) and extensive exome sequencing (∼80×) of 10,000 individuals [19]. In Asia, the 1KJPN panel was constructed by sequencing 1070 Japanese individuals (∼32×) [20]. Moreover, the WGS analysis of the Wellderly cohort has reported the association of healthy aging with reduced susceptibility to Alzheimer’s and coronary artery disease [21].

The Han Chinese constitute the world’s largest ethnic group [22]. In a recent joint investigation of major depressive disorders (CONVERGE), the genomes of 11,670 Han women were sequenced as a control group [23]. With a sequencing depth of 1.7×, this large-scale study of genetic variation among the Han Chinese reported a catalog of 25,057,223 variants. A most recent project by BGI-Shenzhen performed WGS at a high depth (∼80×) in 90 Han individuals, and observed over 7 million (M) novel, low-frequency variants [24]. In view of nearly one fifth of the world’s population [22], a large-scale whole genome study by deep sequencing has been expected to provide an important resource for studying the genetic basis of the disease in Han population.

In 2016, the Chinese Academy of Sciences (CAS) launched the Precision Medicine Initiative (CASPMI) project. The aims of Phase I of this project include: (i) generating a reference genome of a northern Han individual (NH1.0) using an integrated approach; (ii) the WGS (25–35×) of 600 samples from the CASPMI cohort; (iii) association analyses based on sequencing and baseline phenotypic data collected from the project’s participants; and (iv) construction of electronic health records (EHR) and genetic reports for the participants.

Here, we report the near completion of phase I of the CASPMI project, the generation of a high-quality northern Han reference genome, together with a comprehensive genetic map that consists of over 28.8 M variants. We observed several highly differentiated variants in folate cycle-related and lipid metabolism-related genes between northern and southern Han Chinese, which suggests the selection from various environmental exposures and life styles especially the diet habit during the evolution of the Han population. The population-specific data we report here will support and benefit future research aimed at providing precision medicine and individualized healthcare.

## Results

### Sequencing and assembly of the NH1.0 genome

The genomic DNA of a northern Han male (originating from Shandong province) was sequenced using various approaches, as listed in Table S1. These combinational applications included: PacBio for single-molecule sequencing with 49.9 × coverage; the 10× Genomics Chromium system, followed by Illumina sequencing at 60.4 × depth; Illumina paired-end sequencing at 79.2×; Illumina mate pair sequencing at 22.9×; and Bionano Saphyr optical mapping with ∼100 × coverage (GSA Accession Number CRA000631).

Using CANU [25] and Supernova [26] to assemble PacBio long reads and 10× Genomics linked reads, respectively, we generated two sets of genome contigs/scaffolds with N50 of 1.74 Mb and 18.56 Mb, respectively (Table S2). Mismatches and small indels of the PacBio contigs were then corrected by comparison with the Illumina paired-end reads (Table S3), resulting in the decrease of 0.002% mismatch rate and 0.099% indel rate, and an increase of 3,440,598 bp in the assembly length after sequence correction.

After aligning the PacBio contigs to the scaffolds of 10× Genomics and filtering for redundancy, two genome sets were merged into one with the scaffold N50 increasing to 30.45 Mb (Table S2). Bionano optical mapping data were then used for hybrid scaffolding to the chromosomal level, resulting in a further improvement of the scaffold N50 to 46.63 Mb, including 5574 scaffolds and a total assembly length of 2.89 Gb (Table 1). We denoted this assembled genome as NH1.0 (GWH Accession Number GWHAAAS00000000). A haplotype of this genome with a scaffold N50 of 2.16 Mb was also constructed, mainly from the 10× Genomics linked reads.

**Table 1:** **Statistics for the sequencing and assembly of four reference genomes**

	**YH2.0****[2]**	**HX1****[6]**	**NH1.0**	**GRCh38****[1]**
Population	Southern Chinese	Southern Chinese	Northern Han Chinese	European

Sequencing methods	HiSeq (fosmid)	PacBio + BioNano	PacBio + 10× Genomics + Bionano	Sanger (BAC + fosmid)

Assembly software	SOAPdenovo	FALCON	CANU + Supernova	NA

Scaffold N50 (Mb)	20.52	21.98	46.63	67.79

Contig N50 (Mb)	0.02	8.33	3.6	56.41

No. of scaffolds	125,643	5367	5574	735

No. of gaps	235,514	10,901	8484	999

PhaseBlock N50 (Mb)	0.48	NA	2.16	NA

Assembly size (bp)	2,911,235,363	2,934,084,193	2,892,287,479	3,209,286,105

*Note*: NA, not available.

Sequence alignment of the NH1.0 scaffolds to the GRCh38 genome showed high collinearity between these two genomes (Figure S1). Among all the 542 raw scaffolds over 10 kb in length in NH1.0, 23 scaffolds showed inconsistent with the reference genome, mainly due to the local misassembly of complex repeat sequences, as confirmed by checking the mapped paired-end reads near the non-conforming regions. These scaffolds were corrected at the misassembly points accordingly.

As shown in Table 1, compared with the two existing individual genomes of Chinese (HX1 and YH2.0) [5], [6], NH1.0 has longer scaffolds (N50 length of 46.63 Mb versus 21.98 Mb for HX1 and 20.52 Mb for YH2.0), which spans 15 chromosome arms with the coverage of more than 85% of the autosomal regions, and improved integrity at the chromosomal level (Figure S2).

A total of 99 sequencing gaps on GRCh38 were completely filled by the NH1.0 genome. The largest filling consisted of 188,143 bp on the chromosome X, and the total length of the filled gaps was 609,822 bp (Figure S2). Furthermore, 749 novel sequences, spanning 4.76 Mb, were identified by comparing the NH1.0 genome with the reference genome GRCh38. Ranging from 2001 bp to 180,551 bp, the average length of the novel sequences identified was 6348 bp.

To evaluate the representativeness of the NH1.0 genome, Illumina paired-end reads from 15 samples (including three Han Chinese) of five populations in 1KGP were mapped to the GRCh38 and NH1.0 genomes. The Mapping rates for most samples to the two genomes were over 99% (Table S4), although the rates to GRCh38 were approximately 0.3%–0.6% higher, indicating a more complete assembly. However, the mismatch rate of Han Chinese (CHB) paired-end reads to NH1.0 was less than that to GRCh38 by approximately 0.04%, suggesting that the NH1.0 genome is more representative to the Chinese population than the reference genome GRCh38.

**Figure 1:**
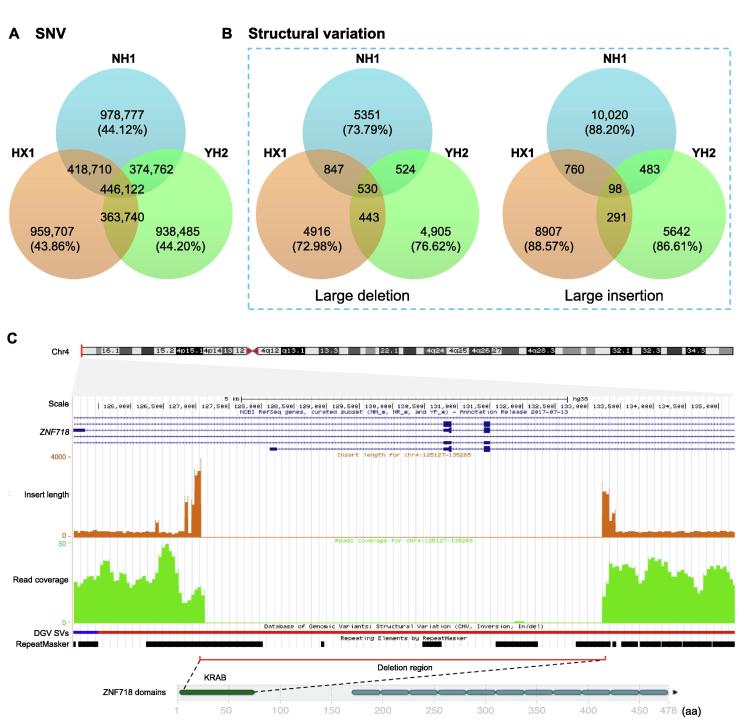
**A comparison of three Chinese reference genomes** **A.** A Venn diagram showing the SNVs present in each of the three Chinese reference genomes and the shared SNVs. **B.** A Venn diagram showing the structural variations shared among the three reference genomes (large deletions on the left and large insertions on the right with SV length >50 bp). **C.** Top, a map of chromosome 4, showing the position of the *ZNF718* gene near the telomere region. Beneath this shows *ZNF718* exons at 5′ end, followed by the mean inner distance (brown) and the coverage (green) of paired-end reads. Both indicate the presence of a homozygous deletion of 6138 bp in the *ZNF718* gene in NH1.0. Below the read coverage is the structural variations shown in the DGV [53], the short blue thick line and the connected dark red thick line indicate the gains and losses, respectively, and the black bars underneath indicate the distribution of repeat elements. Bottom, the domain structure diagram of the protein encoded by *ZNF718* showing that the genomic deletion (red line) results in generation of a truncated *ZNF718* protein lacking the KRAB domain (dark green). SNV, single nucleotide variant; DGV, Database of Genomic Variants.

Comparing to the human reference genome, a total of 2,218,371 single nucleotide variants (SNVs), 378,750 small indels (<50 bp), and 18,613 structural variations (SVs, ≥50 bp) were detected in NH1.0. This northern Han genome also shares 39.0% and 37.0% SNVs with the HX1 and YH2.0 genomes, respectively (Figure 1A), and a total of 55.9% SNVs were observed in the two southern Chinese genomes. By contrast, most structural variations were found to be individual-specific, *i.e.*, 73.79% of the deletions and 88.20% of the insertions in the NH1.0 genome were not found in either of the HX1 and YH2.0 genomes (Figure 1B). With 2.2% of the deletions and 3.3% of the insertions found in exonic regions, most of the identified structural variants (53.2% of the deletions and 66.6% of the insertions) occur in repeat regions (Table S5).

Some of the structural variations were identified with putative functional significance. For example, a homozygous deletion of a 6.1 kb fragment (Figure 1C) encompassing two exons of *ZNF718* was detected. This region was inside a large copy number variation (CNV) of 103 kb, which was recently reported from a diabetes study in the Mexican population [27]. Interestingly, this deletion results in a truncated gene product with the loss of the entire Krueppel-associated box (KRAB) domain, which functions as a transcriptional repressor when tethered to template DNA via a DNA-binding domain [28]. Further investigations to illustrate the deleterious metabolic effects of this homozygous deletion for the sequenced individual, particularly concerning metabolism-related phenotypes, are under way.

### Sequencing and variation analysis in the CASPMI cohort

To investigate genetic variations in the Chinese population, we performed Illumina paired-end WGS (25–35×, Figure S3) on 597 participants of the CASPMI cohort. These individuals come from nine ethnic groups of 30 provinces and autonomous regions (Figure S4). Among them, 455 samples were categorized as northern Han (NH) and southern Han (SH) according to the self-reported ancestry of each participant, and each group of NH and SH consisted of 339 and 116 individuals, respectively (Table S6, and details in Methods).

A total of 24.85 M SNVs and 3.85 M small indels (<50 bp, Table 2) were identified using the Genome Analysis Toolkit (GATK, version 3.5) [29]. Among them, 5.69 M (22.9%) SNVs and 1.27 M indels (33.0%) were common types [allele frequency (AF) ≥5%] and 90.9% of the variants were located in intergenic and intronic regions. Furthermore, 153,884 non-synonymous SNVs and 1588 frameshift indels were detected, and a portion of these variants (10.4% of non-synonymous SNVs and 8.2% of frameshift indels) had AF equal or larger than 5% (Table 3). In mitochondria, 1211 variants were detected, among which 897 (74.1%) were localized in genic regions (Table S7 and Figure S5). To evaluate the accuracy of SNV calling, 890 variants were randomly selected for Sanger sequencing, and 882 (99.1%) were verified (Table S8). In addition, a high-quality haplotype reference of SNVs and short indels was constructed using various phasing approaches.

**Table 2:** **Allele frequency and genomic features of the SNVs and indels identified in the CASPMI cohort**

**Type**	**AF**	**Exonic**	**Splicing**	**ncRNA** **exonic**	**ncRNA** **intronic**	**ncRNA** **splicing**	**5′UTR**	**3′UTR**	**Intronic**	**Up-stream**	**Down-stream**	**Intergenic**	**Total**
SNV	≥50%	11,445	51	5749	105,509	32	2969	13,805	664,216	11,693	12,061	1,088,588	1,916,118
	5%–50%	23,613	65	11,660	199,759	67	6021	27,056	1,343,806	23,236	24,117	2,119,311	3,778,711
	0.5%–5%	25,422	115	8763	144,622	58	5977	24,531	1,025,511	18,148	18,337	1,492,106	2,763,590
	<0.5%	201,609	1595	51,408	872,202	280	41,586	157,899	6,221,982	113,756	109,551	8,619,679	16,391,547
	All	262,089	1826	77,580	1,322,092	437	56,553	223,291	9,255,515	166,833	164,066	13,319,684	24,849,966

Indel	≥50%	347	65	713	17,654	3	467	2778	114,668	2217	2400	173,161	314,473
	5%–50%	651	40	1813	52,435	9	935	7771	366,652	6798	7523	512,111	956,738
	0.5%–5%	1080	43	1594	41,482	13	1058	6936	289,865	5520	5684	393,381	746,656
	<0.5%	7940	458	5213	102,642	45	4264	21,859	702,803	14,524	14,846	958,744	1,833,338
	All	10,018	606	9333	214,213	70	6724	39,344	1,473,988	29,059	30,453	2,037,397	3,851,205

*Note*: SNV, single-nucleotide variant; AF, allele frequency.

**Table 3:** **Statistics of SNVs and indels in the coding regions identified in the CASPMI cohort**

**Type**	**AF**	**Frameshift insertion**	**Frameshift deletion**	**Non-frame-shift insertion**	**Non-frame-shift deletion**	**Nonsynonymous** **SNV**	**Synonymous** **SNV**	**Stop gain**	**Stop loss**	**Unknown**	**Total**
SNV	≥50%	NA	NA	NA	NA	5146	6029	19	4	247	11,445
	5%–50%	NA	NA	NA	NA	10,806	12,320	98	10	379	23,613
	0.5%–5%	NA	NA	NA	NA	13,593	11,345	168	11	305	25,422
	<0.5%	NA	NA	NA	NA	124,339	72,302	2597	103	2268	201,609
	All	NA	NA	NA	NA	153,884	101,996	2882	128	3199	262,089

Indel	≥50%	43	47	84	87	NA	NA	2	0	84	347
	5%–50%	87	149	150	228	NA	NA	7	2	28	651
	0.5%–5%	173	262	164	448	NA	NA	11	0	22	1080
	<0.5%	1285	2945	869	2534	NA	NA	161	6	140	7940
	All	1588	3403	1267	3297	NA	NA	181	8	274	10,018

*Note*: NA, not applicable.

**Figure 2:**
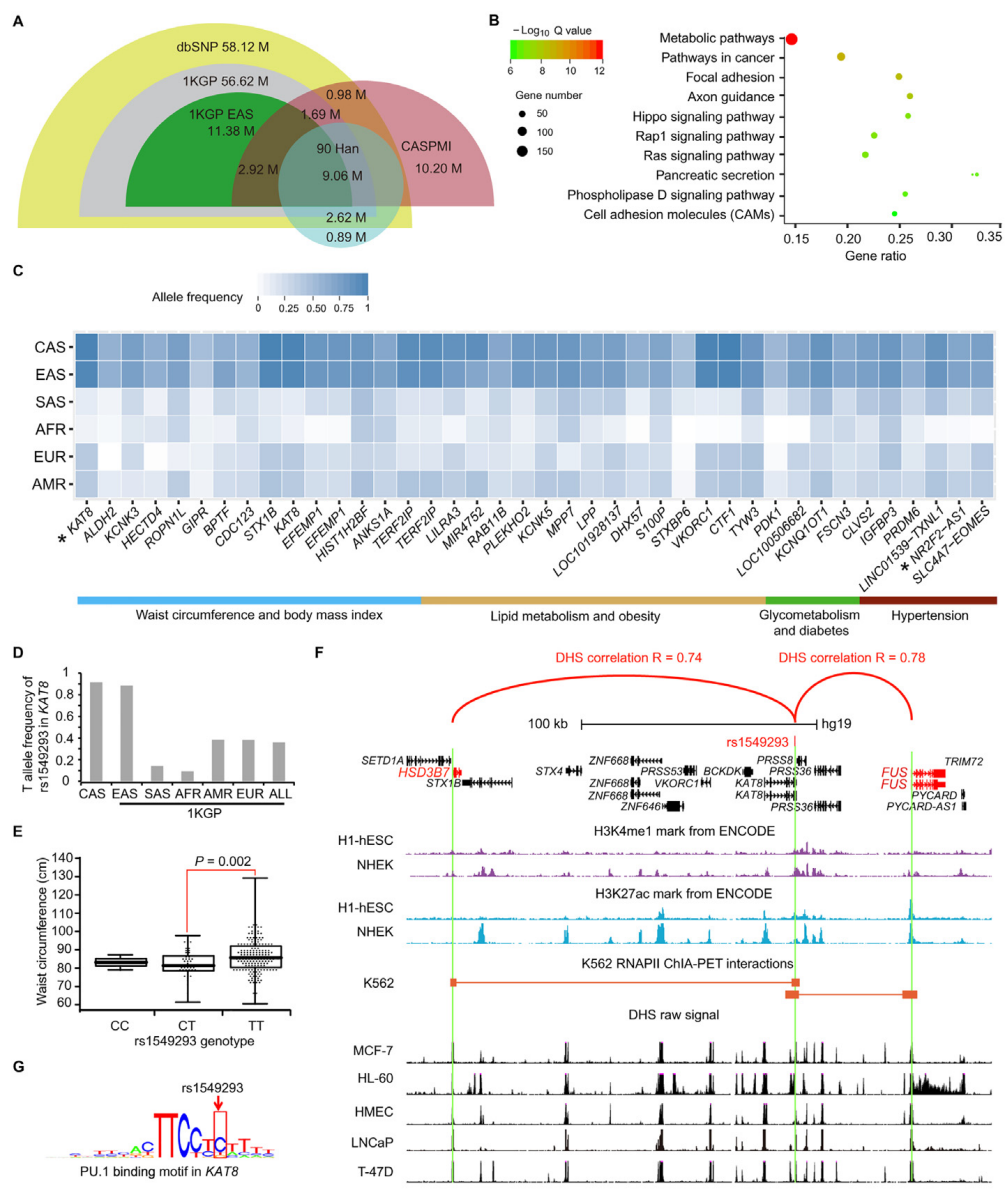
**SNV identification among projects and metabolism-related rs1549293 in *KAT8*** **A.** A comparison of SNVs found in the CASPMI project (pink) with those present in the dbSNP (olive green), 1KGP (gray), 1KGP EAS (green), and the 90 Han Chinese genome study (light blue) [24]. **B.** The enrichment of KEGG pathways for genes with a high frequency of SNPs in the hfCAS-EAS dataset (a group of SNPs with relatively high frequencies in both CASPMI cohort and 1KGP EAS). X-axis represents the ratio of the number of queried genes to the number of total genes involved in each pathway (gene ratio), and y-axis shows the enriched KEGG pathways. The color scale represents Q values (log_10_-transformed) for each enriched pathway (hypergeometric test) and the dot size indicates the number of genes involved in a particular process or pathway. **C.** Genes (shown in x-axis) that are associated with the metabolism-related traits (colored bars underneath) and contain overlapping SNPs present in both hfCAS-EAS dataset and GWAS Catalog. Blue squares in different intensities illustrate frequencies of each SNP in the six populations shown on y-axis. CAS indicates participants of the CASPMI cohort in this study, while EAS, SAS, AFR, EUR, and AMR refer to the respective populations in 1KGP. Genes examined in the current study are indicated using asterisks. **D.** Frequency distribution of the rs1549293-T allele in the aforementioned populations. **E.** Association of waist circumference with different rs1549293 genotypes present in males of the CASPMI cohort (*P* = 0.002, *t*-test). **F.** The interaction of rs1549293 with *HSD3B7* and *FUS* (red arcs) as revealed in various cell types by correlation assays of DHS (black peaks) and ChIA-PET (brick red lines stopping at squares), forming each of 145 kb and 54 kb chromatin interactions, respectively, via recruiting transcription factors PU.1 [36]. The locus where rs1549293 resides is enriched with both H3K4me1 (purple) and H3K27ac (blue) modifications, suggesting an enhancer function of this region. **G.** rs1549293 is localized in a PU.1 binding motif. The affinity for PU.1 binding appears to be weaker with the presence of the T allele [38]. CASPMI, Chinese Academy of Sciences Precision Medicine Initiative; 1KGP, 1000 Genomes Project; EAS, east Asian; hfCAS-EAS, relatively high-frequency SNPs of the CASPMI cohort shared with 1KGP EAS; SAS, South Asian; AFR, African; EUR, European; AMR, Admixed American; DHS, DNase I hypersensitive site.

As illustrated in Figure 2A, as well as Figures S6 and S7, the sample size and sequencing depth of present cohort study enabled us to identify remarkably a total of 10.20 M SNVs and 1.55 M indels that were not listed in dbSNP (7.6% of version 149) nor in 1KGP (version Aug-2015). Although most of these SNVs and indels have allele frequencies below 5%, such a large quantity of novel variants will help to inform and support population and health-related studies in China and globally.

### Population-specific SNPs and metabolism-related annotation

To further characterize population-specific variants from the CASPMI cohort, firstly, we compared the frequencies of the SNPs and indels from our samples to those of different populations from 1KGP. As the Chinese populations, especially northern residents, share the greatest similarity with other East Asian (EAS) people [13], we used the genomes of South Asians, Africans, Native Americans, and Europeans as outlying groups to analyze the frequencies of population variations. By screening for frequency differences that were at least 0.3 greater than those in the outlying groups, we identified 55,271 SNPs and 6774 indels in our samples. We named this set of population-specific variations hfCAS-EAS, standing for the relatively high-frequency SNPs of the CASPMI cohort, which shared with the EAS of 1KGP.

Next, we performed KEGG pathway enrichment analysis of the hfCAS-EAS dataset (Figure 2B). The metabolic pathways of the top enrichment were of particular interest, since one aim of the CASPMI project was to discover genetic correlations of metabolic diseases in an attempt to benefit personal healthcare in this cohort. We then checked the hfCAS-EAS variants that were also present in the GWAS Catalog, and identified 253 SNPs involving 276 genes that are associated with 213 traits or diseases (Table S9). In particular, among these significant SNPs, 40 SNPs in 38 genes correlated with various metabolism-related traits and diseases, including waist circumference, BMI, lipid metabolism, obesity, hypertension, glycometabolism, and diabetes (Figure 2C).

The above results caught our attention, since by comparing the prevalence rate of 8.4% for metabolic syndrome in Chinese males (35–44 years old) from an earlier national report [30], an increased rate of 17.9% in the NH and SH males of the CASPMI cohort was observed, of whom 80% were under the age of 45 (Table S10). Thus, we screened the 40 SNPs that were associated with metabolism in the GWAS Catalog and also present in the hfCAS-EAS dataset (Figure 2C) to identify variants that are corresponding to available phenotypes in our baseline dataset collected from the project’s participants (see Methods). With the discovered 17 SNPs, we conducted a quantitative association analysis employing phenotypic data including waist circumference, fasting plasma glucose levels, and blood pressure, *etc*. (Table 4). In terms of the eight physiological traits or diseases that were investigated, we found that rs1549293 in *KAT8* significantly associated with the waist circumference in males and rs2398162 in *NR2F2-AS1* correlated to hypertension mainly in females (red in Table 4 and asterisks in Figure 2C).

**Table 4:** **Phenotype correlation of the 17 SNPs associated with the metabolic-related traits in the CASPMI cohort**

**Trait**	**SNP**	**Effect allele**	**Major allele**	**Allele frequency**						**Region**	**Gene**	**Annotation data**	**Phenotype association** **(*P* value)**		
				**CAS**	**EAS**	**SAS**	**AFR**	**AMR**	**EUR**				**Total**	**Male**	**Female**
Waist circumference	rs13210323	A	C	0.64	0.69	0.31	0.34	0.31	0.28	Intronic	*ANKS1A*	DHS	0.1073	0.3253	0.361
	rs1549293*	T	T	0.92	0.88	0.14	0.09	0.38	0.38	Intronic	*KAT8*	DHS + ChIA-PET	0.791	0.0093	0.5406
	rs3791679	A	G	0.78	0.77	0.25	0.03	0.24	0.23	Intronic	*EFEMP1*	DHS	0.5242	0.2793	0.446
	rs806794	A	G	0.77	0.76	0.45	0.38	0.45	0.30	3_prime_UTR	*HIST1H2BF*	DHS	0.5319	0.8388	0.3953

Type 2 diabetes	rs11257655	T	T	0.58	0.54	0.24	0.24	0.26	0.23	Intergenic	*CDC123* *CAMK1D*	DHS	0.5517	0.3214	0.55
	rs231356	T	T	0.79	0.79	0.48	0.25	0.43	0.30	ncRNA exonic	*KCNQ1OT1*	DHS	0.5996	0.6583	0.8272
	rs62481355	T	T	0.65	0.69	0.32	0.01	0.25	0.31	Intergenic	*LOC100506682* *GCC1*	DHS	0.7108	0.4669	0.9738
	rs806215	C	C	0.63	0.66	0.26	0.27	0.24	0.22	Intronic	*FSCN3*	DHS	0.8	0.5613	0.9468

Fasting plasma glucose	rs733331	A	A	0.52	0.52	0.18	0.00	0.15	0.04	Intergenic	*PDK1* *RAPGEF4-AS1*	DHS	0.6071	0.6828	0.8547

Hypertension	rs2398162*	A	G	0.65	0.67	0.28	0.04	0.34	0.21	ncRNA intronic	*NR2F2-AS1*	DHS	0.0256	0.6531	0.0071

Systolic blood pressure	rs820430	A	G	0.70	0.67	0.38	0.02	0.37	0.39	Intergenic	*SLC4A7* *EOMES*	DHS	0.6159	0.6138	0.1146
	rs13359291	A	A	0.62	0.59	0.29	0.14	0.27	0.17	Intronic	*PRDM6*	DHS	0.8001	0.8163	0.763

Systolic blood pressure (cigarette smoking interaction)	rs1792738	G	G	0.79	0.78	0.44	0.05	0.43	0.34	Intergenic	*LINC01539* *TXNL1*	DHS	0.1913	0.1317	0.4146

Diastolic blood pressure	rs820430	A	G	0.70	0.67	0.38	0.02	0.37	0.39	Intergenic	*SLC4A7* *EOMES*	DHS	0.8312	0.4117	0.4473

Triglycerides	rs11649653	G	G	0.93	0.90	0.17	0.02	0.41	0.39	Intergenic	*CTF1* *FBXL19-AS1*	DHS	0.6986	0.6136	0.2415

HDL cholesterol	rs759819	C	C	0.81	0.72	0.29	0.07	0.36	0.31	Intergenic	*LILRA3* *LILRA5*	DHS	0.3521	0.1906	0.9844
	rs2967605	T	T	0.64	0.60	0.30	0.22	0.24	0.19	Downstream gene	*RAB11B*	DHS	0.1471	0.3878	0.2987
	rs386000	C	C	0.84	0.65	0.15	0.17	0.43	0.19	Intergenic	*MIR4752* *LILRA3*	DHS	0.76	0.9099	0.6911

*Note*: Association analysis was performed using PLINK toolset. SNPs significantly associated with the phenotypes are put in red (*P* < 0.05). CAS, CASPMI cohort participants in the current study; EAS, East Asian; SAS, South Asian; AFR, African; AMR, Admixed American; EUR, European; HDL, high-density lipoprotein; DHS, DNase hypersensitive site; ChIA-PET, chromatin interaction analysis with paired-end tag sequencing.

As shown in Figure 2D and Table 4, the frequency for T-allele of rs1549293 in *KAT8* (associated with susceptibility to a wider waist) varies dramatically among populations and is high in the CASPMI cohort and East Asians, which reached the highest as 92% in our hfCAS-EAS dataset. In particular, this SNP demonstrated a significant association with larger waist circumferences in the 246 males of our cohort (*P* value = 0.0093, Figure 2E and Table 4). Moreover, within the CASPMI cohort, northern men carrying this SNP, particularly the TT genotype, had significantly larger waist measurements than southern males (Table S11). Considering the general discrepancy in body build between northern and southern Chinese, *i.e.*, significantly taller and larger body mass in northerners [31], the association of the T allele with wider waist measurements implied the potential link to population-based differentiation in the physique.

As illustrated in Figure 2F, the region where rs1549293 resides was a potential enhancer as shown by its H3K4Me1 and H3K27Ac modification from ENCODE data [32].We then searched DNase I hypersensitive sites (DHSs), which showed possible transcription activities in the genome, across a total of 79 cell types [33]. In the genomic locus covering rs1549293, the chromosomal interactions with *FUS* and *HSD3B7* were observed to form each of 54 kb and 145 kb complexes, suggesting a regulatory role by this SNP to these obesity-associated genes (Figure 2F, Pearson correlation coefficient r is 0.78 and 0.74, respectively) [34], [35]. Supporting evidence also came from the polymerase II chromatin interaction analysis with paired-end tag sequencing (ChIA-PET) data [36], which suggested that rs1549293 located in an enhancer that regulated the activities of both promoters of *FUS* and *HSD3B7*. Moreover, this locus was reported as an eQTL to regulate the expression of *HSD3B7* in the Genotype-Tissue Expression (GTEx) project [37]. Importantly, the interaction between rs1549293 and each of these two genes appeared to be mediated by PU.1 transcription factor as demonstrated by a genome-wide study on the binding sites of transcription factors [38]. A significantly weaker affinity was generated when the T allele was present in the motif (Figure 2G). Taken together, we proposed that rs1549293 in *KAT8* correlated to larger waist circumference in the northern males of the CASPMI cohort via the enhancer region containing this SNP, in which the weaker binding to PU.1 by the T-allele may alter the expression of the obesity-associated genes *FUS* and *HSD3B7*.

In addition, we analyzed the genomic features of the SNPs in the hfCAS-EAS dataset based on DNase hypersensitive site (DHS) profiles from 79 cell lines. Among these 55,271 variants, 1751 variants were mapped to the distal DNase I hypersensitive sites which were assigned to 2738 genes genome-wide with an average distance of 228 kb between the SNP and genes, indicating a long-range regulatory role of these variants to the genes through chromatin interactions (Table S12). Moreover, 116 of these connections were further validated by the ChIA-PET data.

By comparing these DHS-related SNPs with the hfCAS-EAS variants that were present in the GWAS Catalog (Tables S9 and S12), eleven of the high-frequency SNPs were shown to be disease- or trait-associated and to locate within the DHSs. Accordingly, the DHSs holding these 11 SNPs were associated with the promoters of their target genes within a ±500 kb region based on their DHS patterns (Table S13). One of these SNPs is rs1549293 in *KAT8* (Figure 2D–G), supporting its putative regulatory role associated with the waist circumference as described above. This list of 11 SNPs provided us with candidates for further phenotype-associated analyses in our cohort, as well as in other populations.

### Genetic differentiation between northern and southern populations

We investigated the genetic structure of the Han population mainly by analyzing the frequency distribution of general variants in NH and SH groups (Table S6). A total of 19,456,897 autosomal SNPs were utilized for the study after filtering for quality control (Methods). As shown in Figure S8, the southern and northern groups were well clustered in the Principle Component Analysis (PCA). In addition, the NH1.0 genome showed to be more representative of northern people in the samples from both CASPMI and 1KGP.

**Figure 3:**
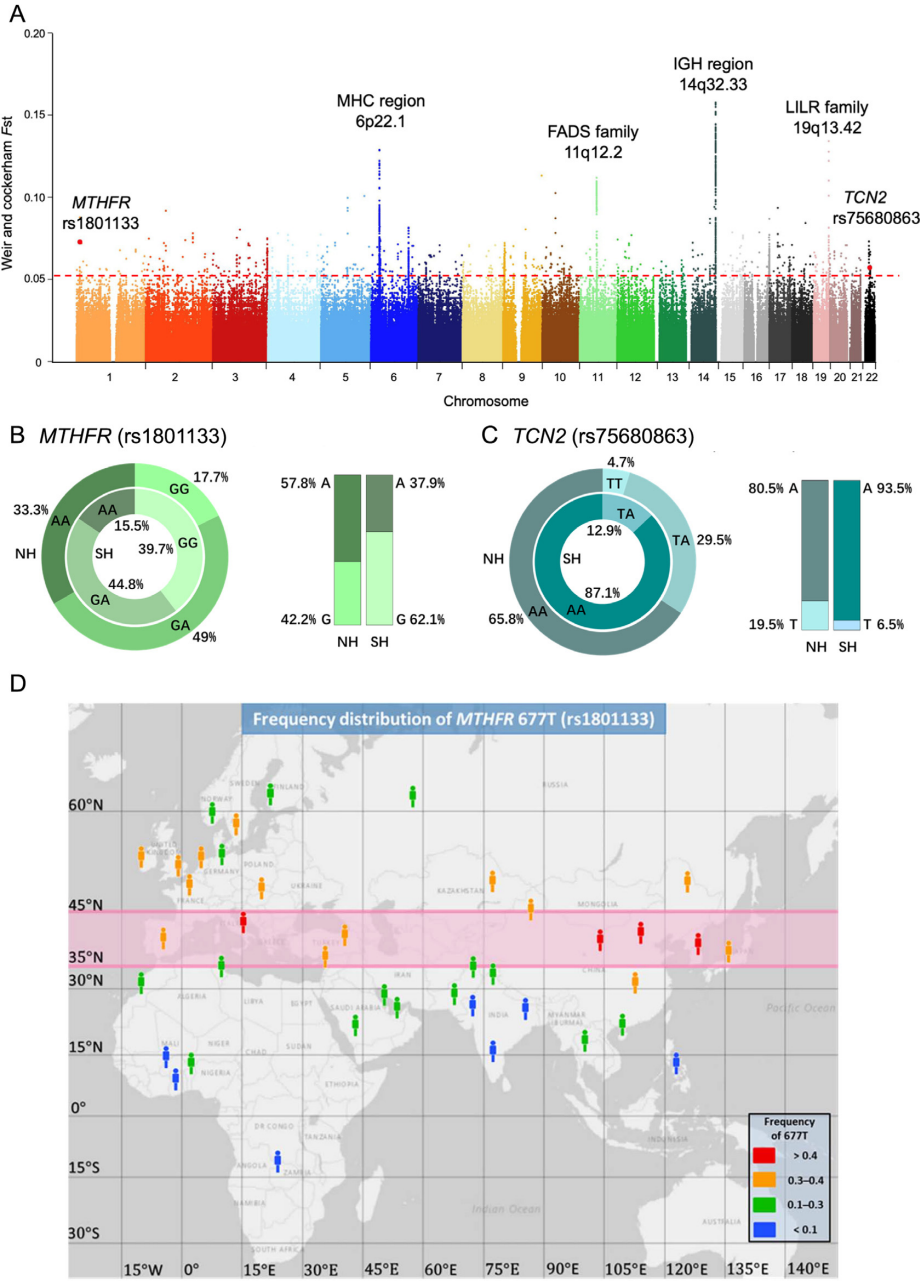
**Genetic differentiation between northern and southern Han populations in the CASPMI cohort** **A.***F*_st_ values between NH and SH populations in the CASPMI cohort. The red dashed horizontal line indicates the *F*_st_ cutoff of ≥0.054. Some top significant regions, genes, and missense SNPs are marked. **B.** Allele frequencies and genotype ratios of *MTHFR* rs1801133 in the NH and SH groups. **C.** Allele frequencies and genotype ratios of *TCN2* rs75680863 in the NH and SH groups. **D.** A relatively high *MTHFR* 667T (rs1801133) belt (colored in red) between latitude 35–45° North. As demonstrated in the map produced by National Geographic Map Maker Interactive (https://mapmaker.nationalgeographic.org/), populations with higher frequencies of 667T are present in the relative central regions of the temperate zone (0.3–0.4 and above, pink belt). The frequency of 667T decreases toward north in Europe and toward south in Africa and Asia (see more details in Table S15), suggesting a selection pressure for higher MTHFR activity in more frigid as well as more tropic area. *F*_st_, the fixation index; NH, northern Han; SH, southern Han.

For the differentiation between the NH and SH based on the analysis of the above SNPs, the average value of the fixation index (*F*_st_) was 0.0015. However, the distribution of *F*_st_ values was highly right-skewed, with a long extending tail (Figure S9), where 1947 SNPs were found with *F*_st_ ≥ 0.054 (empirical *P* < 10^−4^, Table S14). Despite the wide distribution of the highest *F*_st_ signals across the genome, several significant peaks were found to be clustered, mainly on chromosomes 6, 11, 14, and 19 (Figure 3A). The highest *F*_st_ signals were clustered on chromosome 14, near genes mostly related to the immunoglobulin heavy locus (IGH) at 14q32.33. The SNPs, which were present within the two peaks on chromosome 6, located mostly within or around the MHC regions, including the most differentiated missense SNP (rs41549014, *F*_st_ = 0.0800) in HLA-A (Table S14). The differentiation of SNPs in immunity-related regions possibly reflected the exposure of populations to diverse environmental conditions, including climate and diet.

The significant SNP cluster on chromosome 11 located in the fatty acid desaturase (FADS) gene family, where 16, 39, and 4 SNPs were in either intronic or in nearby intergenic regions of the genes *FADS1*, *FADS2*, and *FADS3*. These genes encoded the enzymes which regulate the unsaturation of fatty acids. One SNP in *FADS2* (rs28456), which may influence phospholipid and arachidonic acid levels and was involved in the immune process, showed the highest *F*_st_ (0.1119) in the FADS family [39]. On chromosome 19, four intronic SNPs of *LILRA3* (Leukocyte immunoglobulin-like receptor subfamily A member 3) were highly differentiated between the two Han groups. This gene has been associated with high-density lipoprotein cholesterol (HDL-C) as reported in several studies [40]. For the peak on 19q13.42, a total of 21 SNPs were found within or near the genes of *LILRA3*, *LILRA5*, *LILRB2*, and *MIR4752* (Figure 3A). Thus, some of these population-differentiated SNPs associated with genes that are involved in the metabolism of fatty acids and cholesterol, providing clues about the genomic factors that might contribute to the observed difference in body build between northern and southern Han populations.

The second most-differentiated missense SNP (rs1801133, *F*_st_ = 0.0729, Figure 3B) was in the locus of *MTHFR* on chromosome 1. This gene encodes methylenetetrahydrofolate reductase in the homocysteine and folate metabolic pathway that provided a carbon donor for the methylation of homocysteine to methionine. SNP rs1801133 was also named as C677T of *MTHFR* in early studies as the A allele (677T) leads to an alanine to valine substitution that largely reduced the enzymatic activity of MTHFR up to 70% in the homozygous state [41]. The frequency of allele A was 57.8% in NH and 37.9% in SH in the CASPMI cohort, which was consistent with previous studies showing lower frequencies of 677T in southern populations, but it appears to be contradictory from results in Europe where a high 677T occurred in southern areas (Table S15). By mapping our population results and other reported frequencies to their geographic locations, we hypothesize that there exists an adaptation zone between 35–45 degree North on Afro-Eurasia continents, where the frequency of 677T was maintained high and decreases both northward and southward from this belt (Figure 3D, see Discussion).

Another differentiated missense variant, rs75680863, which is located in the gene *TCN2* on chromosome 22, is also involved in the folate metabolism pathway (Figure 3C). The T allele of this SNP was reported as an East Asia-specific polymorphism and to provide a protective effect against congenital heart defects, as compared to the A allele. The T allele of this SNP had a frequency of 0.195 in NH in our dataset, and a frequency of only 0.065 in SH group, which is similar to the frequency previously reported in the southern Chinese [42].

### Mutational signatures of novel singletons and the population distribution

**Figure 4:**
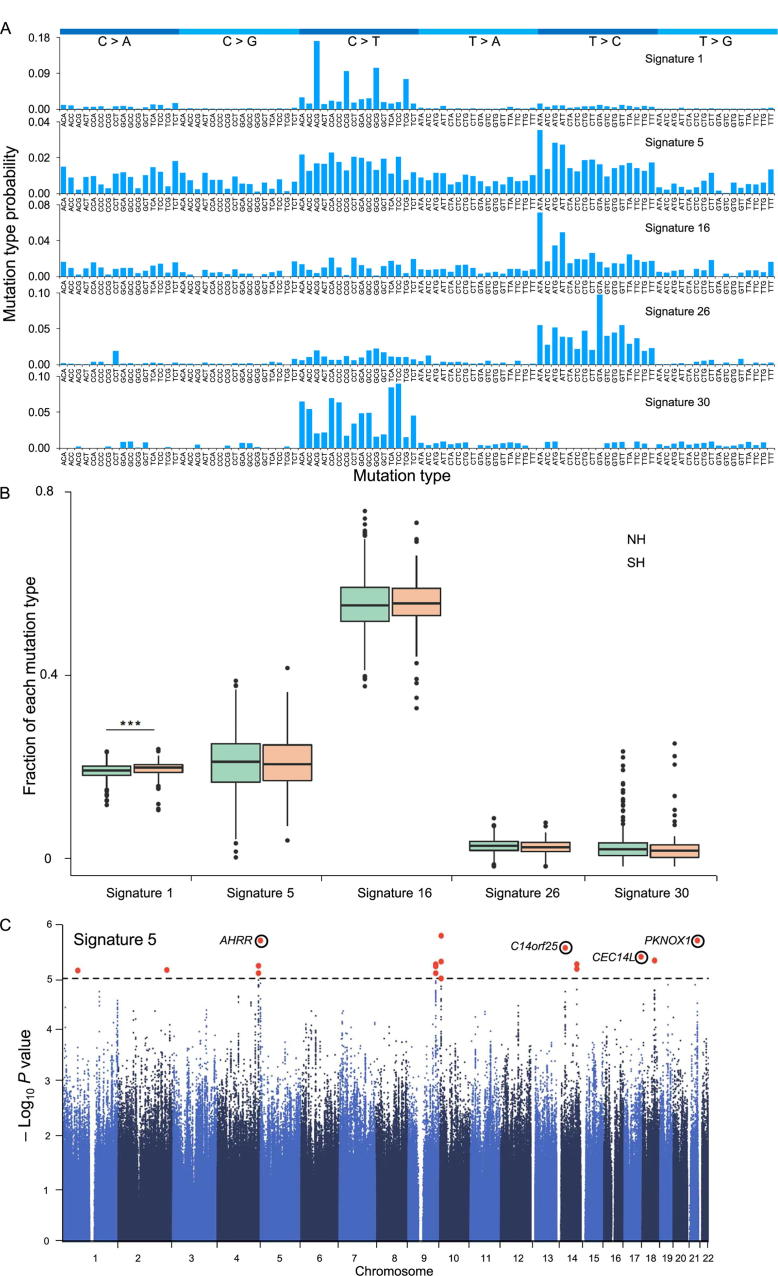
**The population distribution of mutational signatures** **A.** Five COSMIC mutation signatures with patterns matching analysis of the novel singletons identified in the CASPMI cohort. The 96 types of trinucleotide mutational contexts are presented on the x axis, and y-axis shows the probability of a specific mutation occurring in such a context. **B.** Distribution of the five aforementioned mutational signatures in the NH and SH groups. Signature 1 showed the most significant difference between these 2 groups (*P* = 0.001, Wilcox rank test). Boxplots show the proportion of each mutational signature in NH (green) and SH (orange) individuals. Whiskers denote the lowest and highest values within 1.5 times the range of the first and third quartiles, respectively; dots represent outliers beyond the whiskers. **C.** SNPs significantly associated with the individual load of COSMIC signature 5. 17 significant SNPs were identified as being associated with the individual load of this signature (*P* < 10^−5^). Dashed horizontal line represents the significance threshold (*P* = 10^−5^). Red dots represent the significant SNPs, and black circles indicate the genes where the significant SNPs reside. COSMIC, the Catalogue of Somatic Mutations in Cancer.

For the large set of novel singletons which were identified as the SNVs that were absent in dbSNP (Version 150) and present in only one individual of the CASPMI population, we analyzed their mutational signatures to explore the possible mechanisms that might drive the generation of segregating variants. First, we assigned the SNVs into 96 classes, according to the mutation sites and their two flanking bases. We then adopted a non-negative matrix factorization (NMF) to identify specific mutational features [43]. As illustrated in Figure 4A, five mutation signatures, representing different mutational processes in the Catalogue of Somatic Mutations in Cancer (COSMIC) were identified. Among them, signature 16, a mutation type that has been reported to segregate in world-wide populations and only found in liver cancer patients in COSMIC [43], [44], showed the highest individual load (Figure 4B, Figure S10). The other two major mutation patterns of signatures 1 and 5 were age-related, which had been previously identified as the most common types of *de novo* germline mutations [45], [46].

Given that the spectrum of germline mutations can be affected by population-specific genetic factors and by environmental exposure [44], we wondered whether any differentiation had occurred in the mutational patterns between the northern and southern Han populations, whose life styles and residing circumstances differ substantially from each other. Among the five COSMIC mutation signatures identified above, a significant difference was observed in signature 1 between the two groups (*P* value = 0.001, Wilcox rank test, Figure S11, Figure 4B). The signature 1 represented the mutations caused by the deamination of cytosine and accumulates in an age-related manner. The higher incidence of this type of the mutations in southern Han people implied that southerners may have a longer generation time, suggesting a larger effective population size [45], [46], [47]. At the individual level, as indicated in Figure 4B, a large proportion of COSMIC signatures 5 and 16 were observed, although the etiology of both mutation patterns remains unknown. Furthermore, signature 5 showed the largest difference across individuals. By taking the load of this mutation type in each individual as a quantitative trait, we then performed a genome-wide association analysis on the COSMIC signature 5 to identify polymorphisms related to this mutation. In total 17 significant SNPs were identified as being associated with the individual load of this signature (Figure 4C, *P* < 10^−5^), including one SNP located in the gene *AHRR* on chromosome 5. *AHRR* encodes a component in the aryl hydrocarbon receptor signaling cascade, which is involved in cell growth, and mutations in *AHRR* have been shown to associate with male infertility [48].

Taken together, we observed a closely similar mutational spectrum between northern and southern Han people. Both of population divergence and individual difference in the load of mutational signatures may be related to the average generation time, and southern Han may have a longer average generation time as previously reported [49].

### Structural variations in the CASPMI cohort

Three methods were used to identify structural variations (SVs, ≥50 bp) in the CASPMI cohort, namely Pindel [50], CREST [51], and Control-FREEC [52]. The deletions identified by Pindel and CREST, and the inversions and insertions derived from CREST, were separately merged according to their breakpoints by at least 50% overlap between sample-specific identifications. In total, 102,663 deletions, 2249 insertions, and 38 inversions were identified among 597 sequenced individuals (Figure S12). Among all the 106,382 SVs, there are 65,732 novel SVs, comparing to dbVar and the Database of Genomic Variants (DGV) [53], [54] (Table S16). In addition, 1432 copy number variants (CNVs) were detected by Control-FREEC, and the frequencies of most CNVs were below 0.05 (Figure S13).

As shown in Figure S12 and Table S16, among all of the structural variations, 97.8% (102,663) were deletions, of which with an allele frequency <0.05 accounted for 88.6% (92,953). In addition, two-thirds of SVs (66.7%, 70,036) had allele frequencies <0.005, indicating that most SVs were rare or very rare in the sequenced population. With respect to the variation size, most identified SVs were below 20 kb, and the largest variant (deletion) reached 48 kb.

Most SVs were located in repetitive regions. In detail, the SNVs, small indels, large deletions, insertions, and inversions overlapped with 48.9%, 63.5%, 70.5%, 77.6%, and 64.1% of the repetitive regions, respectively (Table S17). In addition, interspersed repeats (also known as mobile elements, MEs) were more commonly found to be associated with most SVs (but not with inversions) than tandem repeats (Table S17), which agreed with previously reported studies [19]. We also found the SVs with complex repetitive elements were generally longer than others, from a comparison of their mean lengths (Table S17).

Finally, to investigate the functional effects of the SVs, genes containing SVs were mapped to the GWAS Catalog. Notably, as listed in Table 5, body mass index and obesity-related traits are among the top 10 significantly enriched terms, which resembled the results of KEGG enrichment analysis for the hfCAS-EAS dataset (Figure 2B). That both the SNVs and SVs identified in this study correlated with metabolism-related traits might be associated with the high prevalence of metabolic syndrome in the males of the CASPMI cohort, as revealed in this project. This possibility would, however, require further investigation.

**Table 5:** **Top 10 enriched traits for the SVs mapped to the GWAS Catalog**

**Term**	**No. of input genes**	**No. of background genes**	***P* value**	**Corrected *P* value (Benjamini-Hochberg)**
Body mass index	41	340	0.0008	0.2190
Schizophrenia	49	441	0.0013	0.2742
Mean platelet volume	11	55	0.0029	0.3312
QT interval	12	68	0.0047	0.3787
Parkinson's disease	15	100	0.0065	0.3787
Nickel levels	8	37	0.0071	0.3787
Adverse response to chemotherapy (neutropenia/leucopenia) (carboplatin)	4	9	0.0074	0.3787
Obesity-related traits	65	689	0.0088	0.3787
Bone mineral density	13	85	0.0095	0.3787
Platelet count	12	78	0.0119	0.3908

## Discussion

### A combination of multiple approaches greatly improved genome assembly

Except for a few circumstances in which traditional Fosmid or BAC libraries are used to improve scaffolding [3], [5], hybrid approaches are also generally used for *de novo* genome assembly of complex organisms. A typical approach is to combine Illumina paired-end sequencing with PacBio sequencing and/or Bionano optical mapping. In this typical approach, such as the assembly of the HX1 genome, sequencing errors of PacBio can be corrected by Illumina reads, while Bionano optical mapping generates longer scaffolds, thus resulting in high-quality contigs that contain less gaps relative to a single approach [6]. Alternatively, Illumina sequencing combined with 10× Genomics library preparation can generate considerably longer scaffolds than PacBio sequencing although more gaps (N-base gaps) may be resulted [4].

To further improve genome assembly in this study, we integrated the approach of 10× Genomics library preparation by Illumina sequencing further with PacBio sequencing, and followed by Bionano mapping (Figure S14). This enabled the construction of longer scaffolds from 10× Genomics sequencing as the main frame of the genome assembly, and PacBio contigs were used to fill gaps by sequence substitutions and to link adjacent scaffolds. This strategy increased our scaffold N50 to 30 Mb. Further integration with Bionano maps resulted in an N50 of >46 Mb, which is more than twice that of previously assembled Chinese genomes (Table 1) and is also better than any combination of Bionano mapping with either 10× scaffolds or with PacBio contigs (Table S2). Accordingly, this integrated strategy greatly increased the scaffold N50 and reduced the number of gaps in assembled sequences.

### Genetic differentiation of Han populations in folate and homocysteine metabolism

As the largest population in the world, Chinese people reside in a vast area. From the most northern to the most southern provincial capitals – Harbin of Heilongjiang province to Haikou of Hainan province – the latitudes differ by 26 degrees (46°N to 20°N), resulting in different adaptations between northern and southern inhabitants to the diverse climates and environments. The most apparent discrepancy between northerners and southerners is perhaps the physique that northern residents, including non-Han ethnic groups, have significantly taller and larger body mass than southern populations [31]. Latitude and environmental variation also lead to remarkable differences in diet among the Chinese. The dominant crop in the north is wheat while that in the south is rice. Moreover, the consumption of vegetables and fruits varies largely in the country. The insufficient ingestion of folate, a soluble B vitamin that is highly enriched in greens, has historically been a severe challenge in certain rural regions of the north and northwest in the country, leading to a high prevalence of various birth defects, such as neural-tube defects [55]. Supplementary folic acid provided in early pregnancy significantly reduces the risks of these birth defects.

Although the overall genetic difference between the northern and southern Han populations is much smaller than that between the Han Chinese and Japanese (*F*_st_ 0.0002–0.0009 versus 0.007–0.008, as reported) [10], some significant genetic differences do exist between these two geographically distinct Han groups, as shown in our study and those of others [22]. Among the near two thousand SNPs identified in our study that have an *F*_st_ value ≥0.054 between northerners and southerners (Figures S8 and S9, Table S14), the missense variants rs1801133 or C677T of *MTHFR*, which is involved in the homocysteine and folate metabolic pathways, caught our immediate attention. This SNP directly correlates to homocysteine levels since the Ala to Val substitution by A allele (677T) results in a much lower MTHFR activity [41]. As an important regulating factor in the folate cycle, as well as an independent risk factor for cardiovascular disease, deficient folate/VB12/VB6 levels and the prevalence of hyperhomocysteinemia (≥10 µM in plasma) have been reported to be more common in northern China [56]. Our finding that the A allele of rs1801133, as well as the AA genotype (677TT), was about 20% higher in the northerners of our cohort (both with *P* value <1 × 10^−3^, Figure 3B) thus provides one interpretation for such observations.

Interestingly, although this significantly higher frequency for 677T of *MTHFR* in the northern group is similar to the results of a few recent studies among Chinese populations, as well as in other Asian populations, 677T is most frequently found in south European populations and its frequency decreases northwardly in Europe (see references of Table S15). Such an apparent opposite decrease tendency in Europe and Asia has been commonly reported, however, without an interpretation [42], [57]. As illustrated in Figure 3D, by mapping the population frequencies of 677T to corresponding geographic locations, the populations with the top most frequencies of 677T, including in relatively southern Europe and in northern regions of China, as well as in Japan and Korea, are actually residing in a latitude zone of 35 to 45 degree North. This led us to raise our hypothesis, the existence of a “comfort” zone between 35 and 45 degree on Afro-Eurasia continents, where 677T frequency is highly adapted (>30%, may reach as high as >45%). From this belt, the frequency decreases both northward and southward including regions of Africa and West Asia although the sample size was limited. One piece of supporting evidence for this hypothesis came from a study in China [58], which showed that comparing to the high 677T in northern area (Hebei), significantly lower frequencies were localized in both the most north region of the northeast (Heilongjiang) and in southern region* (Hainan) (Table S15).

It remains unknown for the driven force that shaped such a narrow but global zone of high 677T in *MTHFR*. In view of the biochemical effect of 677T, as well as the role of *MTHFR* in cycles of folate and homocysteine [59], it is a little easier to understand or imagine how a bi-directional reduction of *MTHFR* 677T formed towards the frigid and the tropic zone. Obviously living in more frigid regions may require full activity of the enzyme to cope with the much less folate intake especially in winter, during the evolution. More interestingly, the decrease range of 677T is larger in the south than that in the north globally, *i.e.*, the higher enzyme activity of *MTHFR* in more tropical regions. This raises another question whether maintaining a suitable blood folate concentration is more critical for early farmers with high but natural folate intake in pre- and early history of agriculture civilization. Nevertheless, if a genomic advantage for 677T allele or physiological benefits from low activity *MTHFR* in this belt of 35–45 degree North, the junctional region between the warm temperate and subtropical zone, awaits for further studies. The related factor(s) that may help answer these questions include environmental conditions [60], nutrition, and dieting including folate intake [61], or genetic differentiation among populations [62]. In particular, it should consider the possible linkage of 677T with other missense loci involved in homocysteine and folate metabolism including rs1801131 (A1298C) also in *MTHFR* and rs1801394 (A66G) in *MTRR*, in which geographic and population diversities were also observed [57].

### Association analysis for waist circumference in males of Chinese populations

We collected phenotypic information on the study participants since the ultimate goal of the CASPMI project is to benefit personal healthcare. It came to our attention that the male participants presented with the metabolic syndrome, as defined by the International Diabetes Federation, were nearly twice that of the national statistics for the average male [63]. This result also supports the earlier report stating that the prevalence of metabolic syndrome with abdominal obesity was significantly higher in males than in female [64].

From a series of phenotype-genotype association analyses, we found that rs1549293, a noncoding SNP of *KAT8* gene, was significantly associated with male waist circumference in our cohort and this variant was also listed in the GWAS Catalog for the same trait (Table 4) [65]. This finding is particularly interesting for several reasons. First, as a highly population-specific SNP, its T allele (particularly the TT genotype) associates with waist width and has the highest frequency of 91.6% in East Asians populations (Figure 2D). More importantly, our stratified phenotype analysis revealed significantly higher waist circumference measurements in northern males with the TT genotype than the men with the same genotype from the South (Table S11), suggesting a possible correlation of this SNP in the physique differentiation of northerners and southerners as stated above. Furthermore, in our association studies on BMI and other metabolism-related phenotypes, the waist circumference results showed the strongest signal, indicating that this measurement is a better predictor than BMI for non-communicable diseases, including cardiovascular disease and type 2 diabetes, as suggested by the World Health Organization. One possible reason might be that, compared to Caucasians, abdominal obesity is the most common type of obesity to occur in Asian populations; thus, waist circumference might better reflect the amount of abdominal adipose tissue and the total body fat in an individual [66]. Finally, taking account of both genetic risks and relatively lacking physical activities, our study thus raises the possibility that northern Han males may be at an increased genetic risk of developing metabolic syndrome, highlighting the potential need to adapt their life styles accordingly, especially for all desk-bound workers.

In summary, in phase I of the CASPMI project, we assembled a high-quality reference genome of the northern Han Chinese. We also provided a comprehensive genetic map containing over 28.8 M variants and a novel population-specific data set hfCAS-EAS resulted from whole genome analyses of near 600 participants. By population-specific analysis between northern and southern individuals, as well as genotype-phenotype association study, our identification of several genes and variants in various metabolism-related pathways demonstrated significant differentiation between northern and southern populations. In our future research, we will enlarge sample size for further identification of rare variations and association studies on more physiological traits in Chinese populations. In view of the current variation identification of the CASPMI cohort which was based on the reference genome hg19, we will release another set of variation data on GRCh38 to increase the data usability of the CASPMI project.

## Materials and methods

### Sample information

This study was performed as a part of the CASPMI Project launched by CAS. Collection and storage of human samples were registered at and approved by the Human Genetic Resources Administration of China (HGRAC). The sample collection protocol was approved by the Institutional Review Board (IRB) of the Beijing Institute of Genomics (BIG), CAS. Participants were from various CAS institutes or offices in Beijing and the community engagement for CASPMI was conducted by lectures and inquiries at each location. Written informed consent was obtained from each participant after community engagement. All data were deposited in the BIG Data Center of BIG, CAS.

A total of 597 individuals participated in this study (246 males and 351 females) with ages between 20 and 60 years old. These participants came from nine ethnic groups of 30 provinces or autonomous regions of China (Figure S4, Table S6). Han individuals were further categorized according to their self-reported ancestry. Individuals with both of their parents reported as being of northern origins were categorized as northern Han (NH) and those who declared non-northern origin of both parents were denoted as Southern Han (SH). A total of 455 samples were identified by either northern or southern ancestry as being 339 NH and 116 SH, respectively (Table S6). All analyses of northern and southern Han groups are based on this categorization.

### Phenotype collection

All phenotypes were collected at the General Hospital of Aviation Industry Corporation of China (AVID). Blood pressure was measured with an automated blood pressure monitor Omron HBP-9021 (OMRON Corporation, Kyoto, Japan) and each measurement was recorded as the average of three times in a seated position after resting for 5 min. Anthropometric measurements, including height, waist circumference, and hip circumference, were obtained using standard protocols with Seca 213 Stadiometer or Seca 203 Body Circumference Measuring Tape (Seca Corporation, Hamburg, Germany). Body weight was examined by an InBody570 Body Composition Analyzer (InBody, Seoul, Korea). Blood samples were collected from participants after an overnight fasting for at least 8 h. Blood tests were processed using a Hitachi Automatic Analyzer 7600 (Hitachi High-Technologies, Tokyo, Japan), including fasting plasma glucose, total cholesterol (TC), triglycerides, low-density lipoprotein cholesterol (LDL-C), and high-density lipoprotein cholesterol (HDL-C).

### Sample preparation and sequencing

For the NH1.0 genome, the fresh blood sample was collected from a healthy male of northern Han. Genomic DNA was extracted and purified using TIANamp Blood DNA kit (Catalog No. DP348-03, TIANGEN Biotech Co., Beijing, China). Two PacBio libraries with the insert size of >10 kb were prepared and then sequenced with P6-C4 reagent kits on PacBio RSII (Pacific Biosciences, Menlo Park, CA). A library with the insert size of 300–500 bp was prepared and sequenced on Illumina HiSeq 3000 (Illumina, San Diego, CA) with 2 × 101 bp read length (Catalog No. FC-410–1003, PE-410-1001, Illumina). Other three mate pair libraries with the insert sizes of 3–5 kb, 5–8 kb, and 8–10 kb were also constructed using Illumina Nextera Mate Pair Sample Prep Kit (Catalog No. FC-132-1001, Illumina) followed by sequencing on HiSeq 3000 with 2 × 101 bp read length. High molecular weight genomic DNA was obtained from fresh blood using MagAttract HMW DNA kit (QIAGEN, Venlo, Netherlands) to prepare a 10× Genomics barcode library according to manufacturer’s protocol. This barcode library was sequenced on Illumina HiSeq X (Illumina) with 2 × 150 bp read length (Catalog No. FC-501-2521, Illumina). Additionally, genome optical mapping data was obtained using Saphyr System (Bionano GENOMICS, San Diego, CA) with nicking enzyme Nt.BspQI according to manufacturer’s protocols.

For population samples, genomic DNA sequencing libraries were prepared using NEXTflex Rapid DNA-seq Kit (Catalog No. 5144-08, Bioo Scientific Corporation, Austin, TX, USA) followed by sequencing on the Illumina HiSeq 3000 or HiSeq X.

### Genome assembly

PacBio long reads and 10× Genomics linked reads were assembled respectively using CANU [25] and Supernova [26] with default options. Mismatches and small indels in PacBio contigs were corrected using Illumina paired-end reads.

To combine the two data sets of assembled genomes, the PacBio contigs over 50 kb were aligned to the scaffolds assembled from 10× Genomics linked reads using MUMMER [67], and sequence overlaps between the two data sets were identified and classified to 8 types (Figure S15). Using in-house Perl/Python scripts, 10× Genomics scaffold sequences were substituted with PacBio contigs in the matched regions where PacBio contigs are fully included, and were linked by the PacBio contigs which were mapped to the ends of two 10× Genomics scaffolds. The PacBio contigs with ambiguous alignment to 10× Genomics scaffolds were excluded in the PacBio-10× merging process to avoid the errors on determining sequence overlaps. Then Bionano optical mapping data was used for hybrid scaffolding using Bionano Solve (V3.0.1) [68]. Finally, the gaps in hybrid scaffolds were filled with Illumina paired-end reads using Gapcloser [69].

### SNV and indel calling and validation

After removing sequencing adapters and trimming consecutive low-quality bases from both the 5′ and 3′ end of the reads using an in-house Perl script, the clean reads were mapped to the reference human genome (hg19) using BWA (V0.7.12) [70] with default parameters. The Picard tool (http://picard.sourceforge.net) was used to sort mapping results to BAM format and mark duplicates of PCR amplification. Then GATK (V3.4) was used for variant calling and filtration. To validate the accuracy of SNVs calling, 890 SNVs were randomly selected, and the fragments contained the SNV loci were amplified by PCR and validated using Sanger sequencing.

### Detection of structural variations

For SVs identification in three Chinese individual genomes, scaffold sequences were aligned to GRCh38 using LASTZ [71], and candidate SVs were obtained using SOAPsv packages. Then SVs were filtered by comparing the ratio of aligned single-end reads to paired-end reads (S/P ratio) as described in a previous study [72].

For SVs identification in the CASPMI cohort, using hg19 as the reference, CREST (V1.0.1), Pindel (V0.2.5b8), and Control-FREEC (V10.6) [52] were used with default parameters. According to the previous studies [50], 10 kb and 50 kb were selected as the max threshold of SV size for Pindel and CREST, respectively, and the SVs longer than 50 bp were kept for further analyses. For each sample, the SVs overlapped more than 80% were merged, and the start and end position were defined as the mean of the start and end positions of each SV, respectively. Across samples, the SVs overlapped more than 50% were merged to achieve non-redundant SVs. The SVs overlapped more than 80% with the gaps in the human genome (hg19) were removed.

### Annotation of genomic variants

Genomic variations were annotated for allele frequencies and associated gene functions using ANNOVAR (version May-11-2017) [73] with the built-in databases, such as RefGene, dbSNP (version 147), 1KGP (version 2015aug), ClinVar (version 20170130), and GWAS Catalog. Pathway enrichment analysis of variation association genes was performed using KOBAS 3.0 [74].

DHS patterns across diverse cell types were used to correlate the distal DHS to the promoter of genes as described by Thurman and the colleagues [33]. The Pearson correlation coefficients (*r*) were calculated between signals of promoter-DHS and distal DHS within ±500 kb. If the coefficient is greater than 0.7, the distal DHS was considered to be correlated with the gene where the promoter DHS located. The H3K4me1, H3K27Ac, and ChIA-PET data were obtained from the UCSC Genome Browser. The DNA features and regulatory elements around the non-coding SNPs were annotated using the online tools regulomeDB [75].

### Haplotype construction

A subset SNVs (with fine-scale recombination map provided by SHAPEIT2) from GATK were extracted as the input of SHAPEIT2 to construct haplotype scaffolds. Subsequently, MVNcall was used to build the haplotype reference panel based on the haplotype scaffolds with default parameters.

### Population differentiation analysis

*F*_st_ between northern and southern Han Chinese populations was calculated based on the autosome SNPs of 455 samples with distinguished ancestry information. After filtering for SNPs with missing rate >50%, a total of 19,456,897 SNPs were retained for further analysis. The SNP-specific Weir and Cockerham *F*_st_ estimator between NH and SH was calculated using VCFtools [76].

### Association analysis

Association analysis was performed by PLINK toolset [77]. For metabolic-trait association analysis, the 10 phenotypes listed in Table 4, including 8 quantitative traits and 2 self-reported diseases, were recruited from the EHR system. The 8 quantitative traits were normalized by the R package. The association test for quantitative traits and case-control was conducted by PLINK linear regression module and logistic regression module, respectively.

For genome-wide association analysis, the quantitative trait was set as the individual load of mutational signature 5. The SNPs with population frequencies larger than 0.05 and with most missing genotype in one sample were finally used for analysis. A threshold of association significance was set to 1 × 10^−7^.

### Mutational signatures identification

For the SNV calling from all individuals, we focused on variants presented only once in entire samples, also known as singletons. The SVs, which were supported by more than 10 reads in each individual and were not presented in dbSNP build 147, were identified as novel singletons.

Each of the novel singletons was classified into 96 possibly mutated trinucleotides, according to the mutation site and its two flanking bases. Then the count matrix of the 96 possibly mutated trinucleotides for all samples was generated. The context of each mutation type was extracted from the human reference genome build hg19. Then, the mutation profiles which were fit to known signatures from COSMIC were estimated by the R package of “MutationalPatterns”.

## Data availability

The raw sequencing data of NH1.0 have been deposited in the Genome Sequence Archive [78] (GSA: CRA000631). The NH1.0 genome sequence has been deposited in the Genome Warehouse (GWH: GWHAAAS00000000), which is publicly accessible at the BIG Data Center (http://bigd.big.ac.cn/gwh). The sequencing data, variant calls, and inferred genotypes for other CASPMI individuals will be available upon request after approval by the Ethical Committee and the Data Access Committee of CASPMI.

## Authors’ contributions

CZ and JX supervised the study. XL (Xin Liu), CZ, JX, FL, and XS designed sample and phenotype collection. XL (Xin Liu), FL, XL (Xi Lu), WZ, XS, DZ, YW, and SS (Shuhui Song) participated in sample and phenotype collection. BZ, YS, WL, ML, and QQ constructed sequencing libraries and generated genomic data. ZD, LM, HQ, WC, JX, HC, XF, NY, SS (Shuo Shi), JZ, JW, YY, QL, YH, LD, and ZZ performed data analysis. CZ, ZD, LM, HQ, WC, BZ, and JX wrote and edited the manuscript. All authors read and approved the final manuscript.

## Competing interests

The authors have declared no competing interests.

## Supplementary material

Supplementary Figure S1 **The top ten longest NH1.0 scaffolds aligned to the GRCh38 genome** NH 1.0 scaffolds (y-axis) were aligned to GRCh38 (x-axis) by MUMmer. The red and blue lines indicate the alignments are in the same strand or in the opposite strand, respectively.

Supplementary Figure S2 **Genome coverage comparison between the reference GRCh38 and three Chinese genomes** Cytobands indicate the GRCh38 genome. Reference genome coverage by de novo assembled scaffolds are shown by three lines above the cytobands, which from the top to bottom stand for HX1 (blue), YH2.0 (orange), and NH1.0 (green), respectively. On green lines, chromosomal arms that were covered by NH1.0 assembly for at least 80% were marked in dark red. Below each cytoband, the histogram shows the position and number of GRCh38 gaps filled by NH1.0 (scale on y-axis).

Supplementary Figure S3 **Distribution of sequencing depth for CASPMI samples** X-axis shows the sequencing depth while corresponding sample numbers were shown on y-axis.

Supplementary Figure S4 **Geographical distribution of the CASPMI cohort participants** Red lines delineate the boundaries of geographical regions in China, which are also marked with letters. White lines indicate the borders of different provinces and autonomous regions. Areas are color coded according to the number of cohort participants where they come from. The northern regions referred to in the current study include NW, N, NE, Shandong province of E, and Henan province of C. Individuals with both of their parents reported as being of northern origins were categorized as NH, and those who declared non-northern origins of both parents were denoted as SH. NE, northeast; N, north; NW, northwest; E, east; C, central; SW, southwest; S, south.

Supplementary Figure S5 **Mitochondrial SNPs in the CASPMI cohort A.** Circular mitochondrial genome. Genes were depicted in different colors based on their positions in the outer ring. The number of samples (log2-transformed) containing minor alleles was illustrated in histogram in the inner ring. **B.** Distribution of the mitochondrial SNPs in the CASPMI individuals. **C.** Allele distribution of 48 mitochondrial SNPs in the CASPMI cohort.

Supplementary Figure S6 **Indels identified from the CASPMI project** A comparison of indels identified in the CASPMI project (pink) with those present in the dbSNP (olive green), 1KGP (gray), 1KGP EAS (green), and the 90 Han Chinese genome study (90 Han, blue) [24]. 1KGP, 1000 Genomes Project; 1KGP EAS, East Asians included in 1KGP.

Supplementary Figure S7 **Allele frequency distribution of known and novel SNVs and indels in the CASPMI cohort A.** Allele frequency distribution of SNVs. **B.** Allele frequency distribution of indels.

Supplementary Figure S8 **PCA plots of 1KGP EAS and the three Chinese reference genomes A.** The first two principle components in PCA plot showing 1KGP EAS (including CHB, CHS, CDX, JPT, and KHV) and the three Chinese reference genomes (NH1.0, YH2.0, and HX1). **B.** The first two principle components in PCA plot showing 1KGP EAS with the Han Chinese populations (CHB and CHS) excluded, the three Chinese reference genomes (NH1.0, YH2.0, and HX1), as well as the northern Han and southern Han in our study (NH-CASPMI and SH-CASPMI). PCA, principal component analysis; CHB, Han Chinese in Beijing; CHS, Southern Han Chinese; CDX, Chinese Dai in Xishuangbanna; JPT, Japanese in Tokyo; KHV, Kinh in Ho Chi Minh City, Vietnam.

Supplementary Figure S9 **Genome-wide empirical distribution of *F*_st_ between northern and southern Han populations** The number of SNPs and *F*_st_ values are shown on Y and X axes, respectively. The 99.99th percentile is indicated using the dashed vertical line. The number of SNPs with *F*_st_ ≥0.054 were shown in the inset.

Supplementary Figure S10 **Individual load of the five COSMIC signatures in the CASPMI cohort** X-axis indicates 597 samples, y-axis indicates the proportion of each of the 5 signatures shown in Figure 4A in the respective individuals. Signatures 1, 5, 16, 16 and 30 are color coded in red, brown, green, blue, and purple, respectively.

Supplementary Figure S11 **Distribution of the sample proportion of the mutation signature 1 in northern (red) and southern (green) Han populations**

Supplementary Figure S12 **SV distribution of the CASPMI cohort A.** SV distribution by length. Each bar represents the counts of three types of SVs within a 500-bp window. **B.** Frequency distribution of SVs showing that more than 90% of SVs occur with allele frequency <0.05, and DEL accounts for more than 90% of all SVs in all ranges. X-axis represents allele frequency interval and y-axis represents the SV counts. SV, structural variation; DEL, deletion; INS, insertion; INV, inversion.

Supplementary Figure S13 **Allele frequency distribution of CNVs in the CASPMI cohort** The y-axis shows the CNV counts with given allele frequency intervals shown on the x-axis. CNV, copy number variation.

Supplementary Figure S14 **Genome assembly workflow for NH1.0**

Supplementary Figure S15 **Overlap classification and merging strategy for PacBio-10× Genomics scaffolds A.** 8 possible types (a–h) of overlapping PacBio-10× Genomics scaffolds. **B.** Merging strategy for PacBio-10× Genomics scaffolds.
