# Supplementary material for: Whole Genome Analyses of Chinese Population and De Novo Assembly of A Northern Han Genome
Source: Genomics Proteomics Bioinformatics. 2019 Sep 5;17(3):229–47. doi: 10.1016/j.gpb.2019.07.002 (PMC6818495; doi:10.1016/j.gpb.2019.07.002)
Supplement: Supplementary Figure S1 — The top ten longest NH1.0 scaffolds aligned to the GRCh38 genome NH 1.0 scaffolds (y-axis) were aligned to GRCh38 (x-axis) by MUMmer. The red and blue lines indicate the alignments are in the same strand or in the opposite strand, respectively. [file mmc1.pptx]

## Slide 1
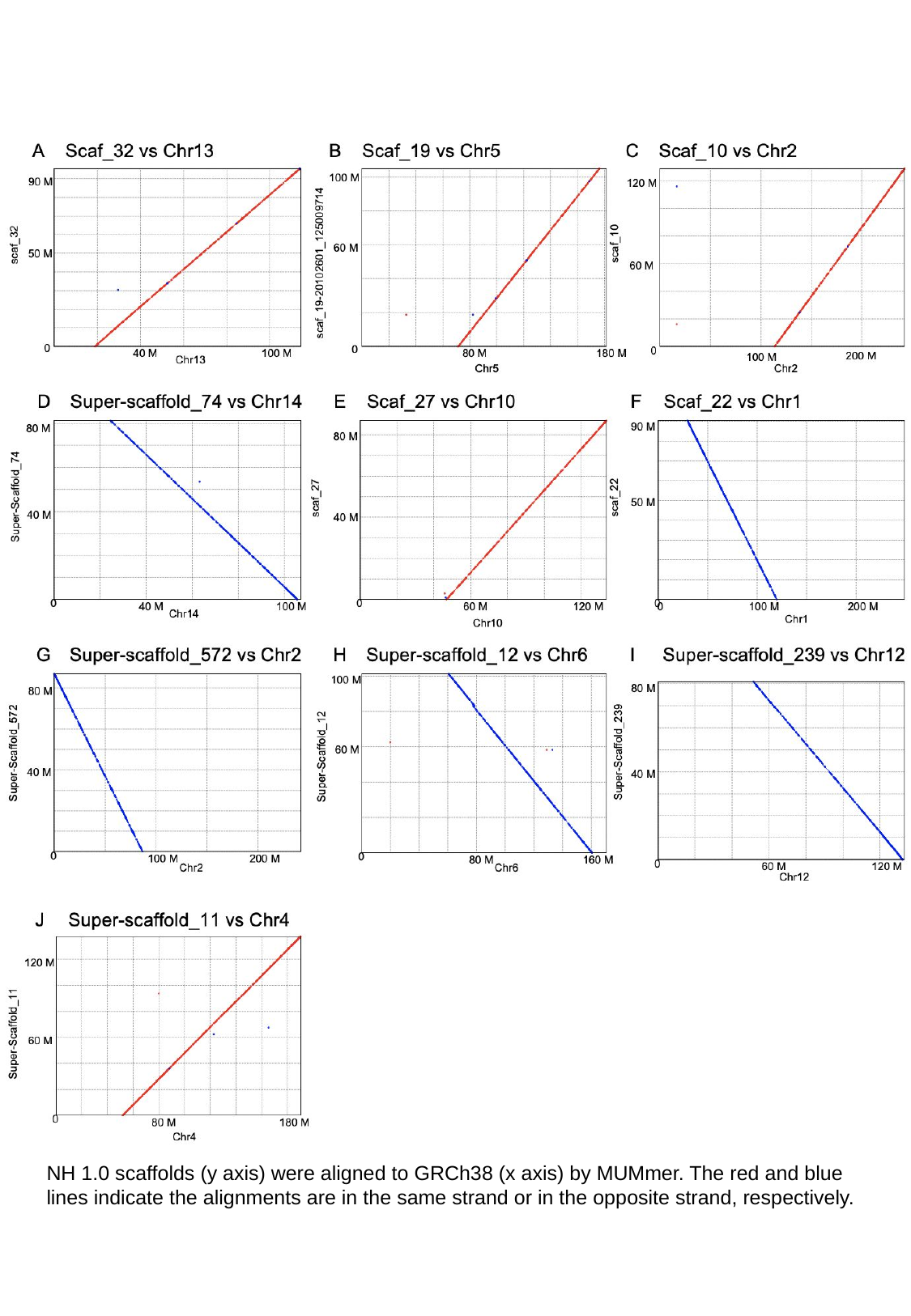

NH 1.0 scaffolds (y axis) were aligned to GRCh38 (x axis) by MUMmer. The red and blue lines indicate the alignments are in the same strand or in the opposite strand, respectively.
