# Supplementary figures and images for: Whole Genome Analyses of Chinese Population and De Novo Assembly of A Northern Han Genome
Source: Genomics Proteomics Bioinformatics. 2019 Sep 5;17(3):229–47. doi: 10.1016/j.gpb.2019.07.002 (PMC6818495; doi:10.1016/j.gpb.2019.07.002)

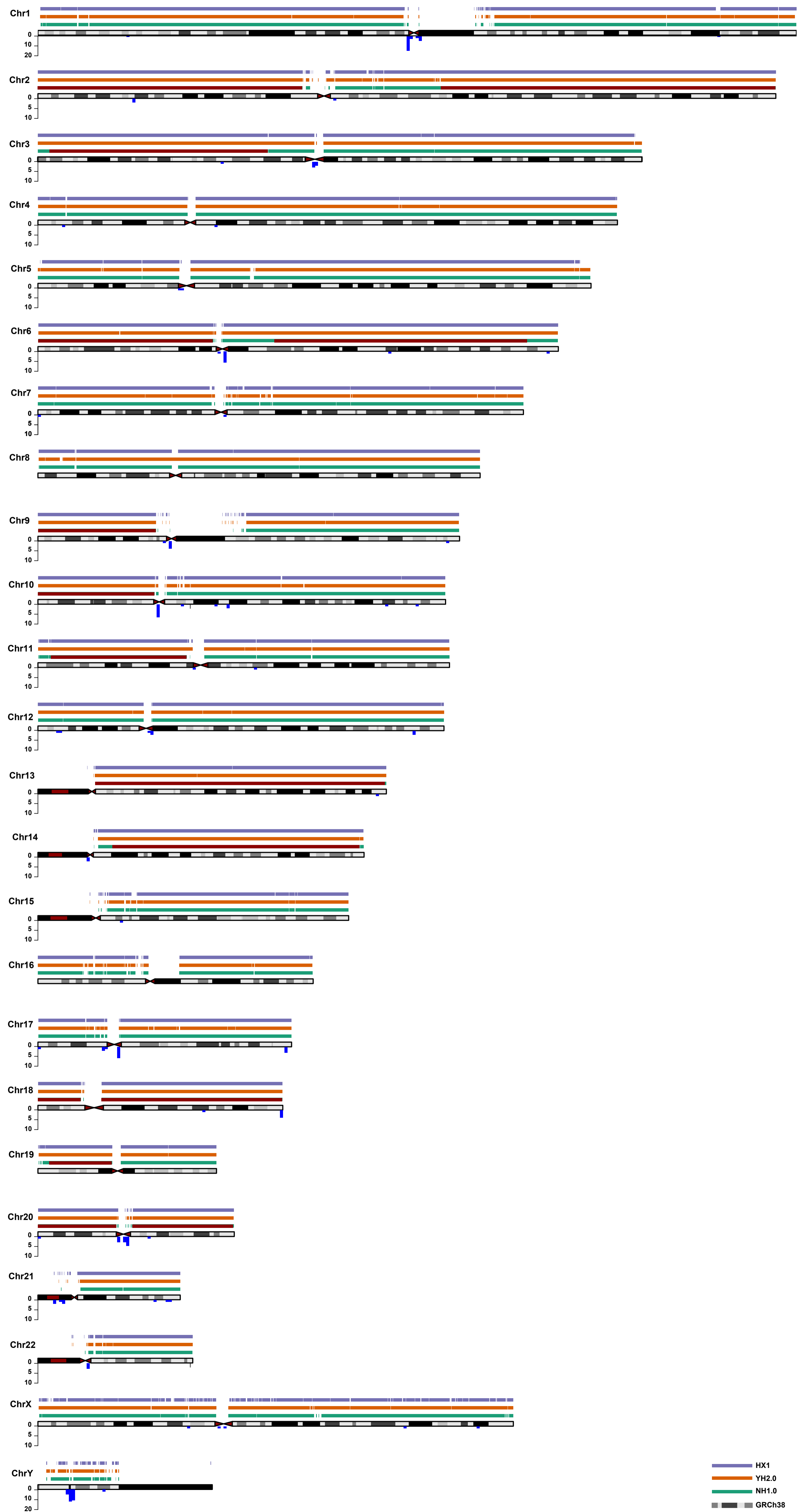

Supplement: Supplementary Figure S2 — Genome coverage comparison between the reference GRCh38 and three Chinese genomes Cytobands indicate the GRCh38 genome. Reference genome coverage by de novo assembled scaffolds are shown by three lines above the cytobands, which from the top to bottom stand for HX1 (blue), YH2.0 (orange), and NH1.0 (green), respectively. On green lines, chromosomal arms that were covered by NH1.0 assembly for at least 80% were marked in dark red. Below each cytoband, the histogram shows the position and number of GRCh38 gaps filled by NH1.0 (scale on y-axis). [file mmc2.pdf]

## Slide 1
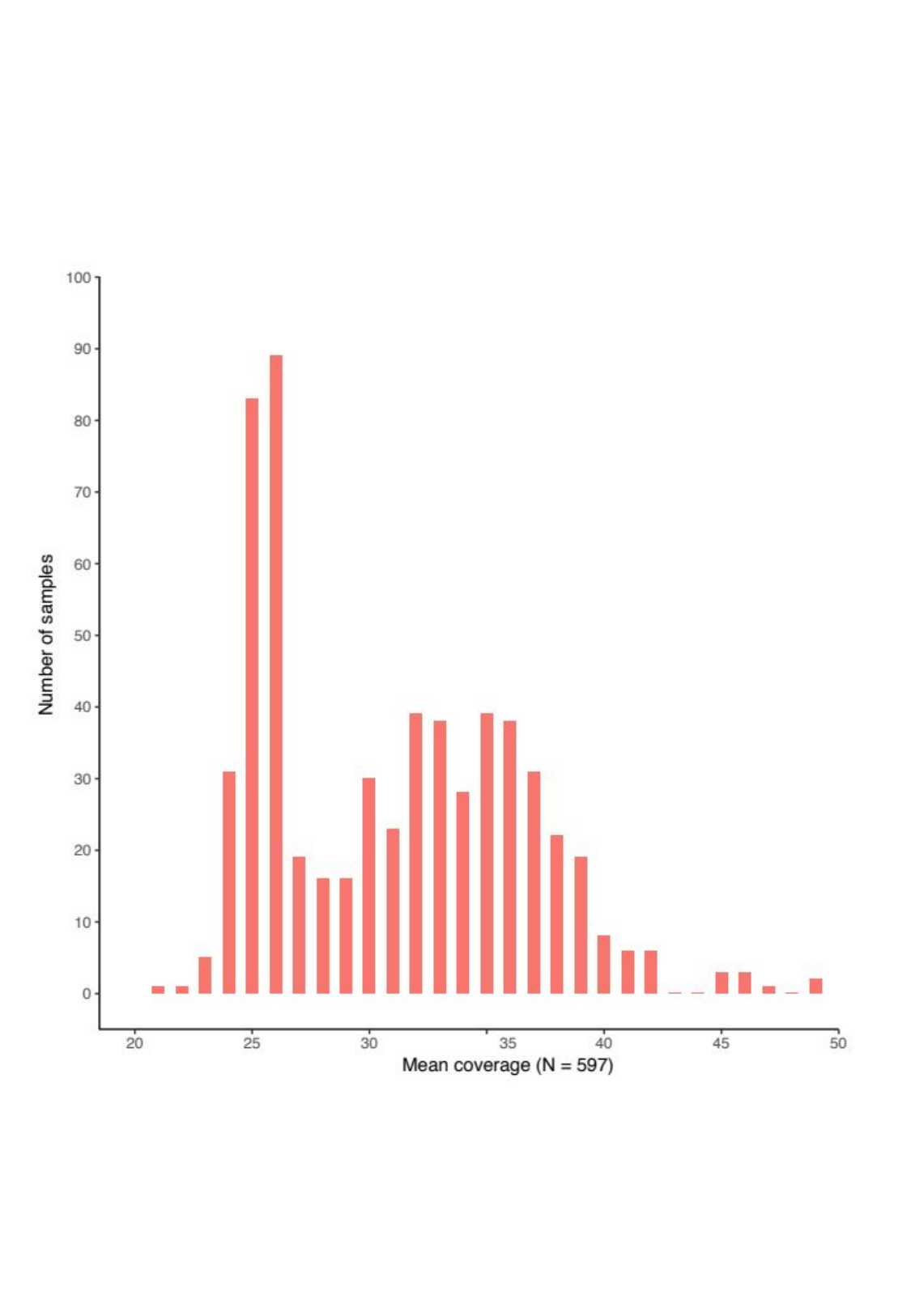

Supplement: Supplementary Figure S3 — Distribution of sequencing depth for CASPMI samples X-axis shows the sequencing depth while corresponding sample numbers were shown on y-axis. [file mmc3.pptx]

## Slide 1
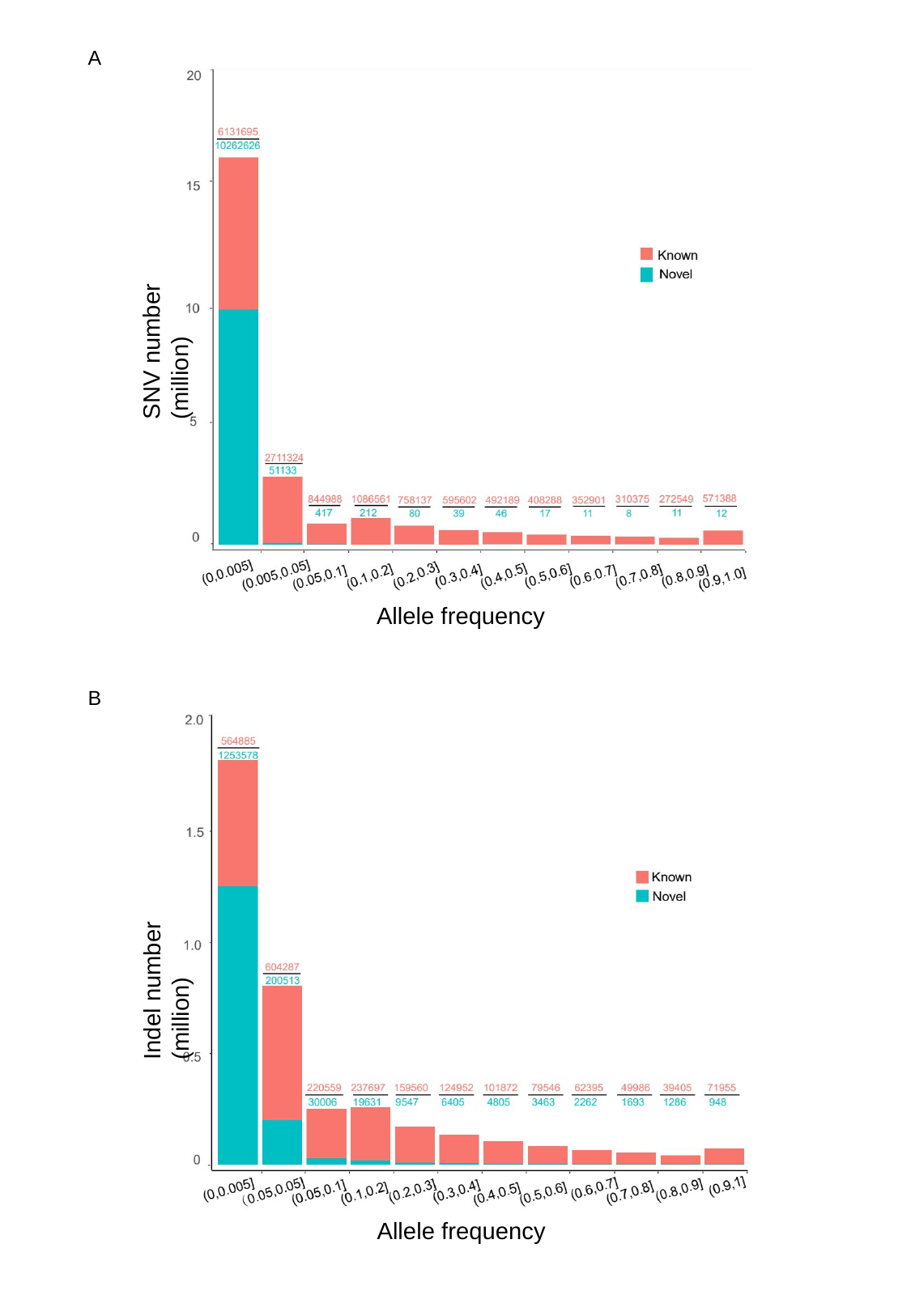

A
SNV number (million)
Allele frequency
B
Indel number (million)
Allele frequency

Supplement: Supplementary Figure S7 — Allele frequency distribution of known and novel SNVs and indels in the CASPMI cohort A. Allele frequency distribution of SNVs. B. Allele frequency distribution of indels. [file mmc7.pptx]

## Slide 1
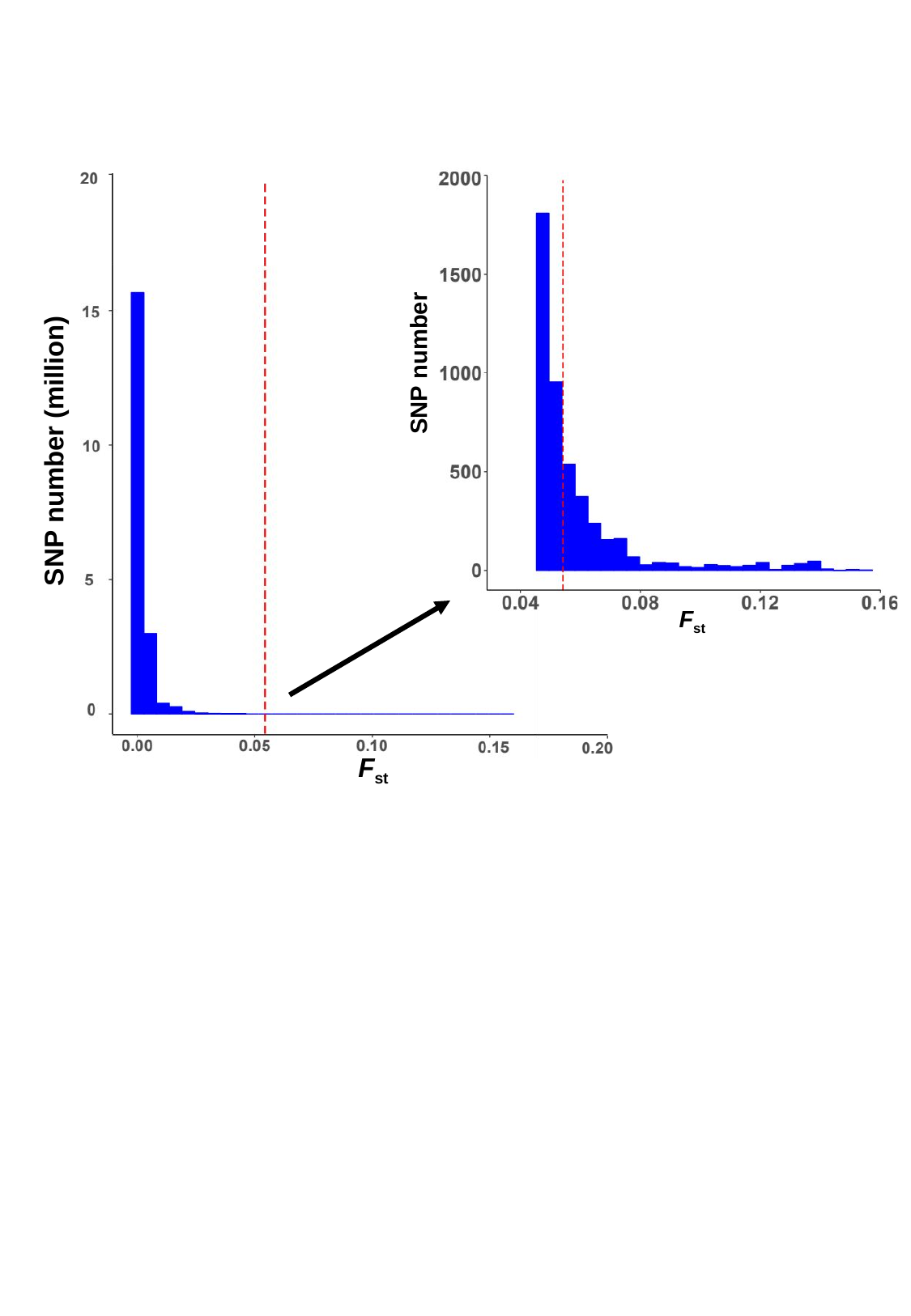

SNP number
SNP number (million)
Fst
Fst

Supplement: Supplementary Figure S9 — Genome-wide empirical distribution of Fst between northern and southern Han populations The number of SNPs and Fst values are shown on Y and X axes, respectively. The 99.99th percentile is indicated using the dashed vertical line. The number of SNPs with Fst ≥0.054 were shown in the inset. [file mmc9.pptx]

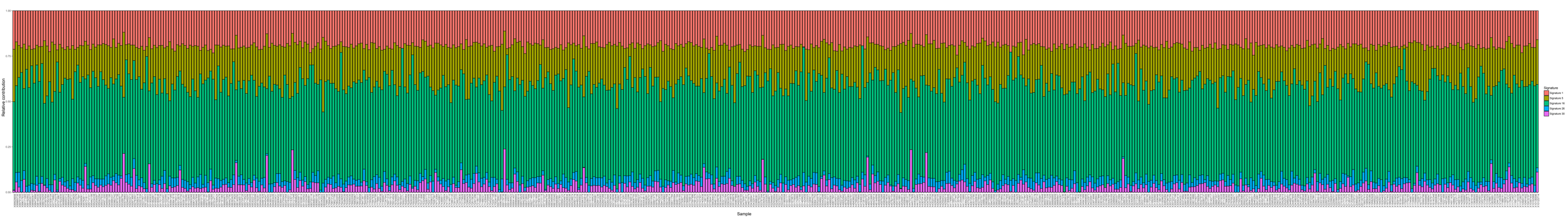

Supplement: Supplementary Figure S10 — Individual load of the five COSMIC signatures in the CASPMI cohort X-axis indicates 597 samples, y-axis indicates the proportion of each of the 5 signatures shown in Figure 4A in the respective individuals. Signatures 1, 5, 16, 16 and 30 are color coded in red, brown, green, blue, and purple, respectively. [file mmc10.pdf]

## Slide 1
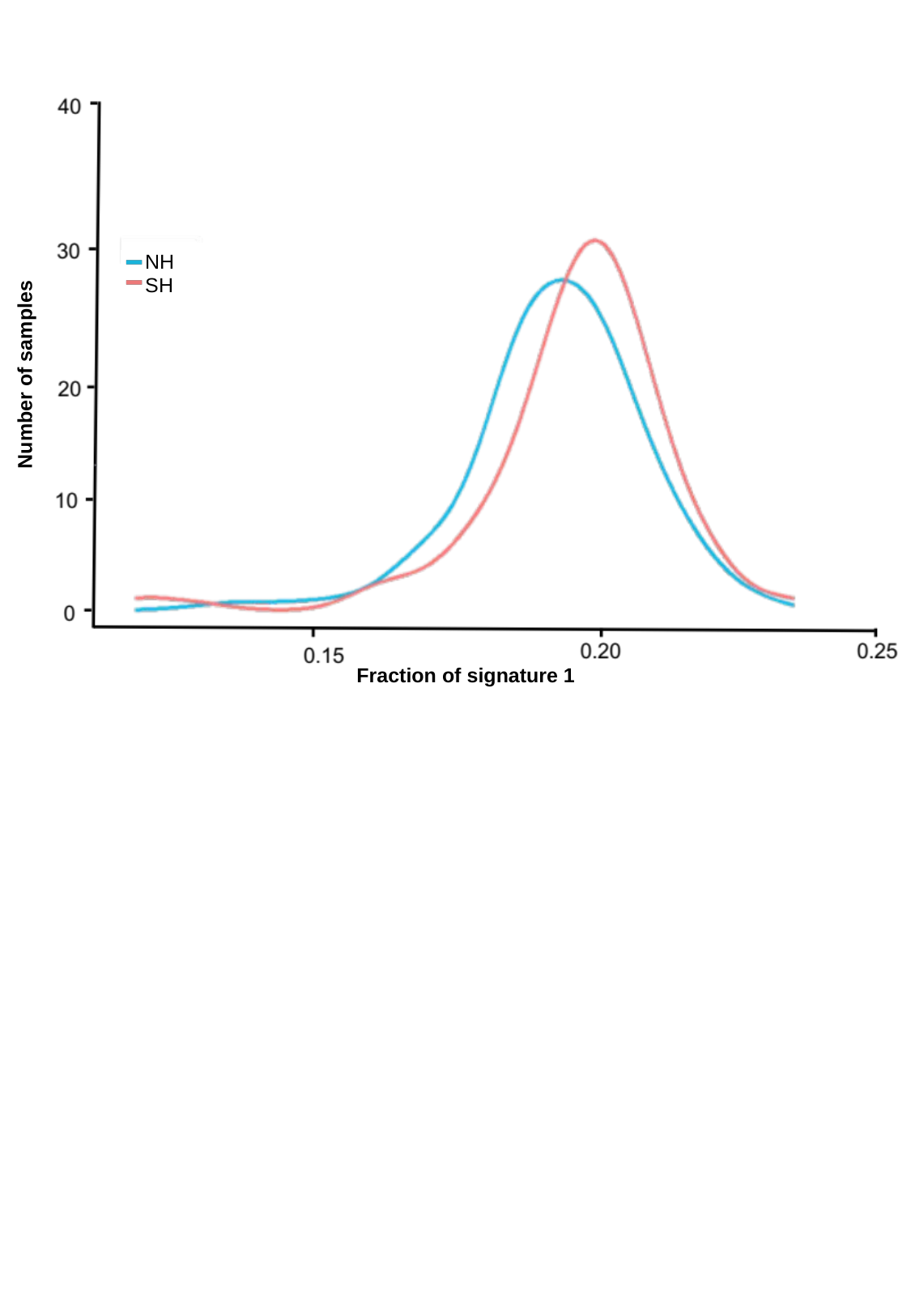

NH
SH
Number of samples
Fraction of signature 1

Supplement: Supplementary Figure S11 — Distribution of the sample proportion of the mutation signature 1 in northern (red) and southern (green) Han populations [file mmc11.pptx]

## Slide 1
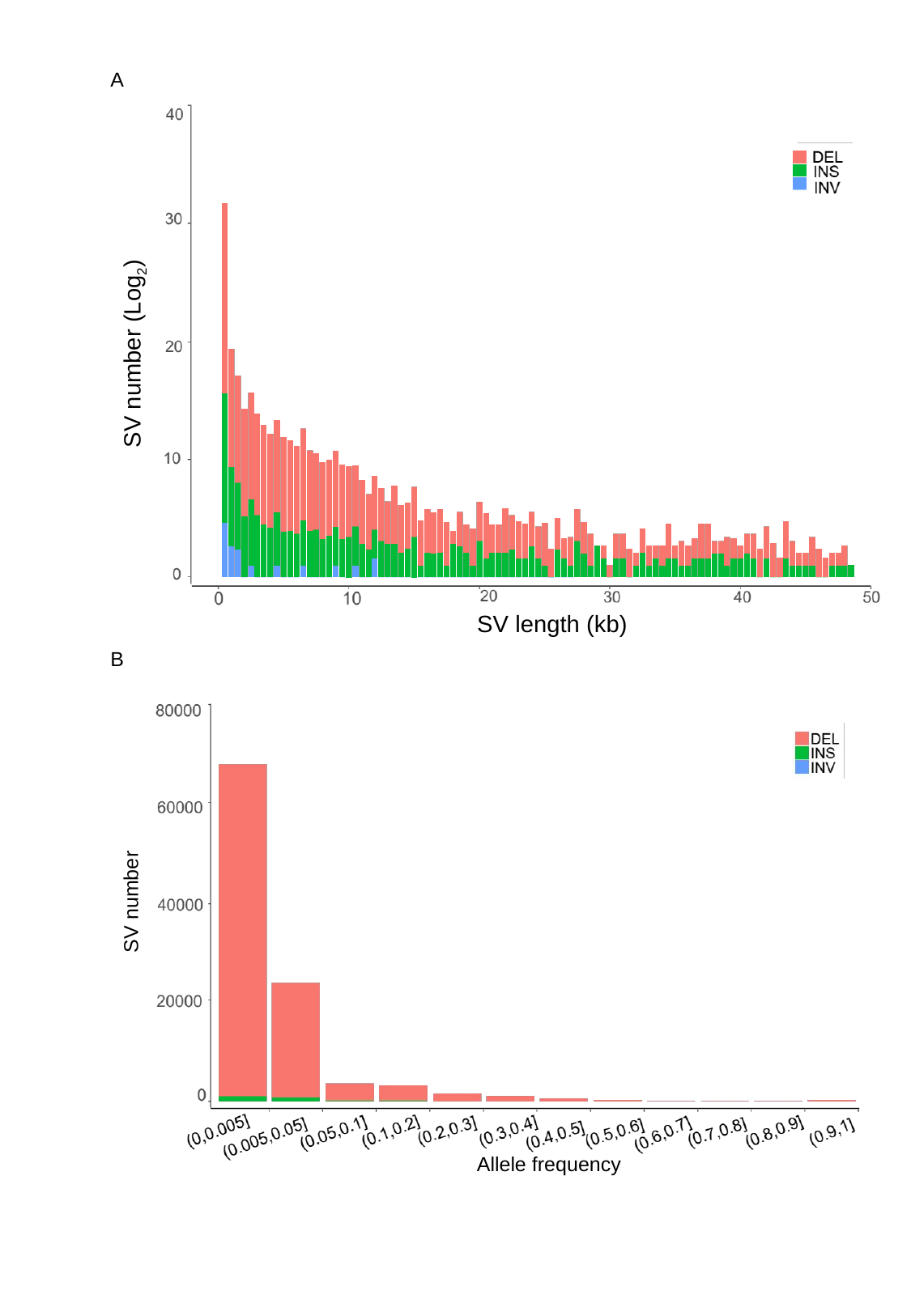

A
SV number (Log2)
SV length (kb)
B
SV number
Allele frequency

Supplement: Supplementary Figure S12 — SV distribution of the CASPMI cohort A. SV distribution by length. Each bar represents the counts of three types of SVs within a 500-bp window. B. Frequency distribution of SVs showing that more than 90% of SVs occur with allele frequency <0.05, and DEL accounts for more than 90% of all SVs in all ranges. X-axis represents allele frequency interval and y-axis represents the SV counts. SV, structural variation; DEL, deletion; INS, insertion; INV, inversion. [file mmc12.pptx]

## Slide 1
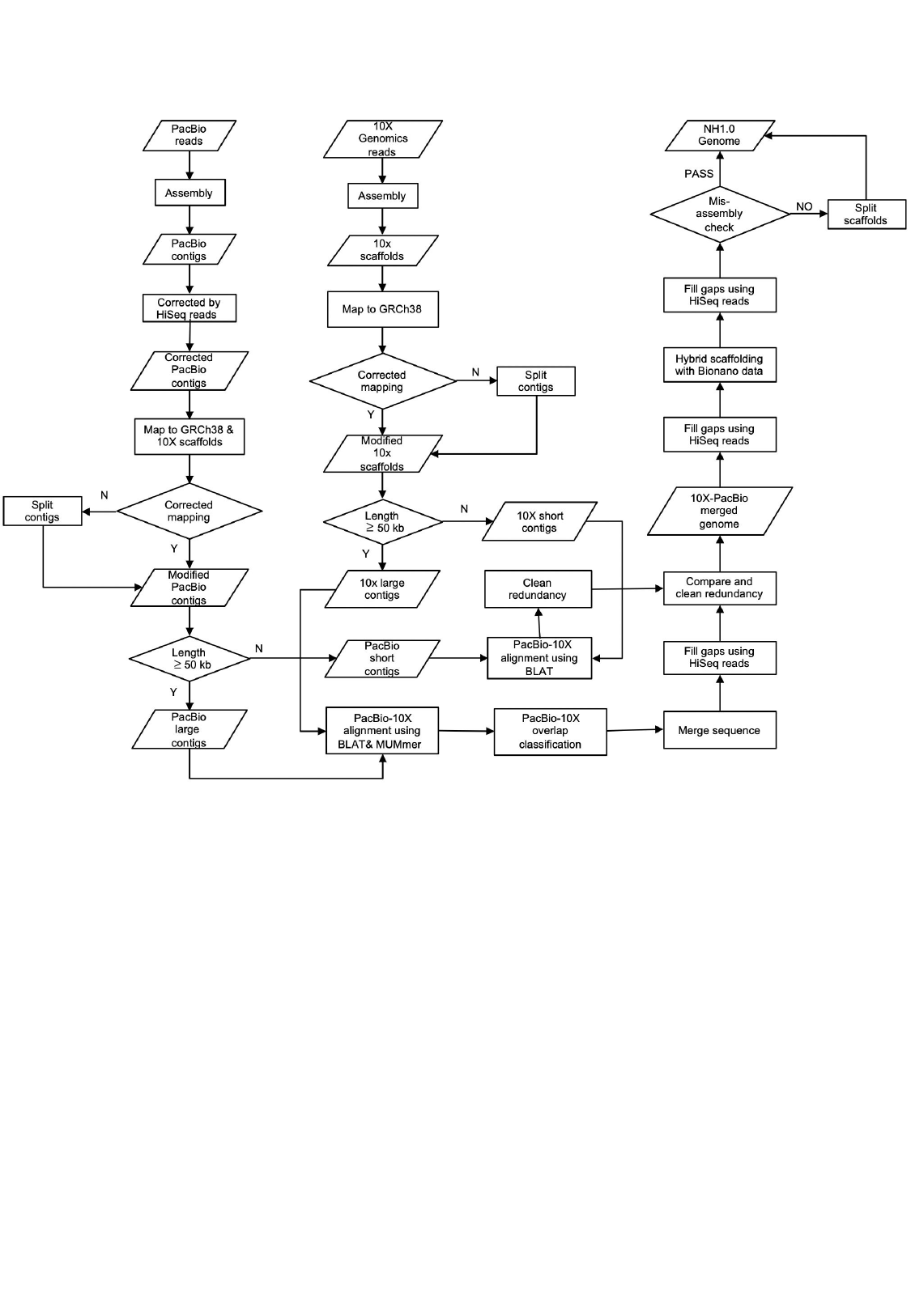

Supplement: Supplementary Figure S14 — Genome assembly workflow for NH1.0 [file mmc14.pptx]

## Slide 1
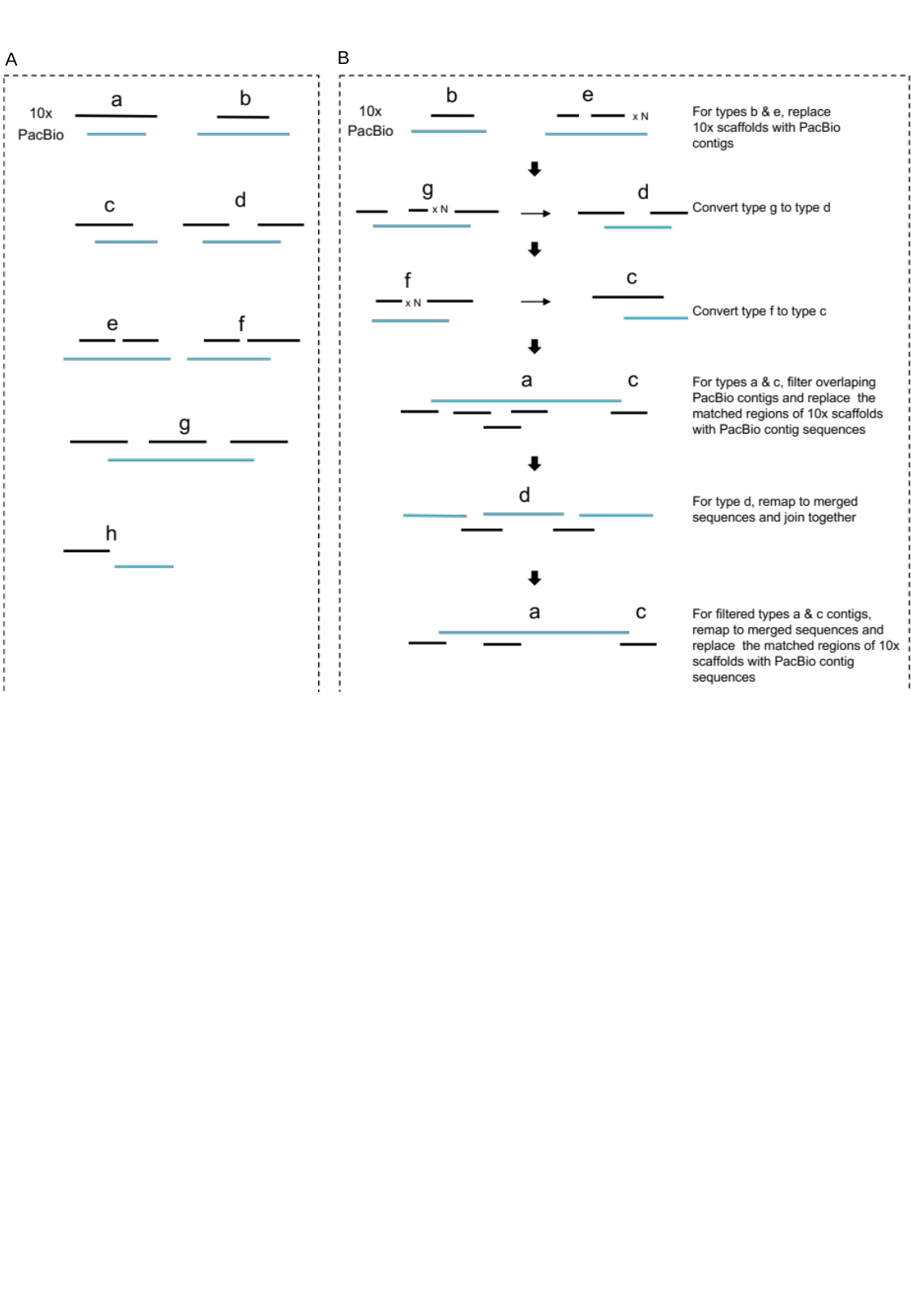

B
A

Supplement: Supplementary Figure S15 — Overlap classification and merging strategy for PacBio-10× Genomics scaffolds A. 8 possible types (a–h) of overlapping PacBio-10× Genomics scaffolds. B. Merging strategy for PacBio-10× Genomics scaffolds. [file mmc15.pptx]
