# Supplementary material for: Whole Genome Analyses of Chinese Population and De Novo Assembly of A Northern Han Genome
Source: Genomics Proteomics Bioinformatics. 2019 Sep 5;17(3):229–47. doi: 10.1016/j.gpb.2019.07.002 (PMC6818495; doi:10.1016/j.gpb.2019.07.002)
Supplement: Supplementary Figure S4 — Geographical distribution of the CASPMI cohort participants Red lines delineate the boundaries of geographical regions in China, which are also marked with letters. White lines indicate the borders of different provinces and autonomous regions. Areas are color coded according to the number of cohort participants where they come from. The northern regions referred to in the current study include NW, N, NE, Shandong province of E, and Henan province of C. Individuals with both of their parents reported as being of northern origins were categorized as NH, and those who declared non-northern origins of both parents were denoted as SH. NE, northeast; N, north; NW, northwest; E, east; C, central; SW, southwest; S, south. [file mmc4.pptx]

## Slide 1
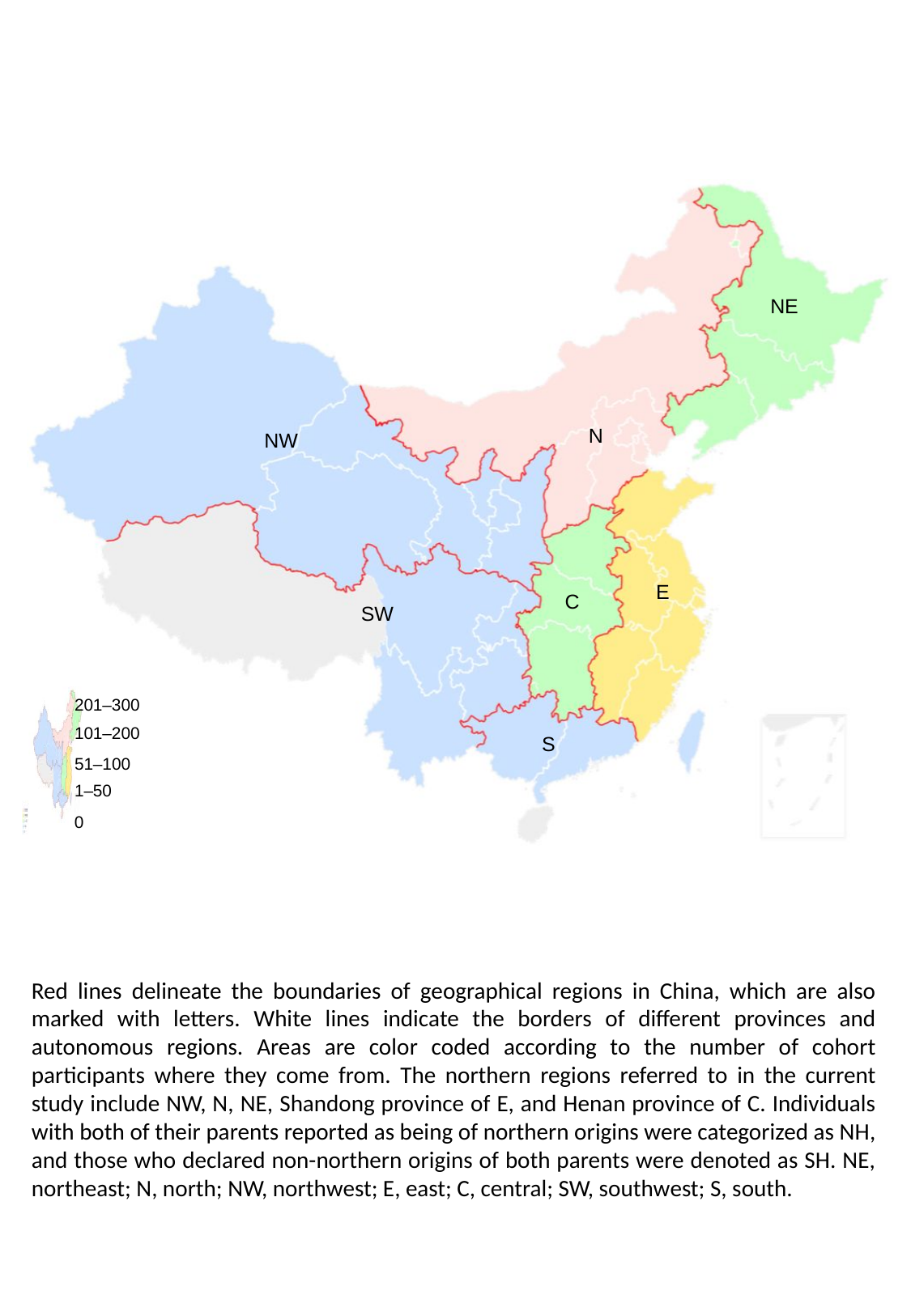

NE
N
NW
E
C
SW
S
201–300
101–200
51–100
1–50
0
Red lines delineate the boundaries of geographical regions in China, which are also marked with letters. White lines indicate the borders of different provinces and autonomous regions. Areas are color coded according to the number of cohort participants where they come from. The northern regions referred to in the current study include NW, N, NE, Shandong province of E, and Henan province of C. Individuals with both of their parents reported as being of northern origins were categorized as NH, and those who declared non-northern origins of both parents were denoted as SH. NE, northeast; N, north; NW, northwest; E, east; C, central; SW, southwest; S, south.
