# Supplementary material for: Whole Genome Analyses of Chinese Population and De Novo Assembly of A Northern Han Genome
Source: Genomics Proteomics Bioinformatics. 2019 Sep 5;17(3):229–47. doi: 10.1016/j.gpb.2019.07.002 (PMC6818495; doi:10.1016/j.gpb.2019.07.002)
Supplement: Supplementary Figure S5 — Mitochondrial SNPs in the CASPMI cohort A. Circular mitochondrial genome. Genes were depicted in different colors based on their positions in the outer ring. The number of samples (log2-transformed) containing minor alleles was illustrated in histogram in the inner ring. B. Distribution of the mitochondrial SNPs in the CASPMI individuals. C. Allele distribution of 48 mitochondrial SNPs in the CASPMI cohort. [file mmc5.pptx]

## Slide 1
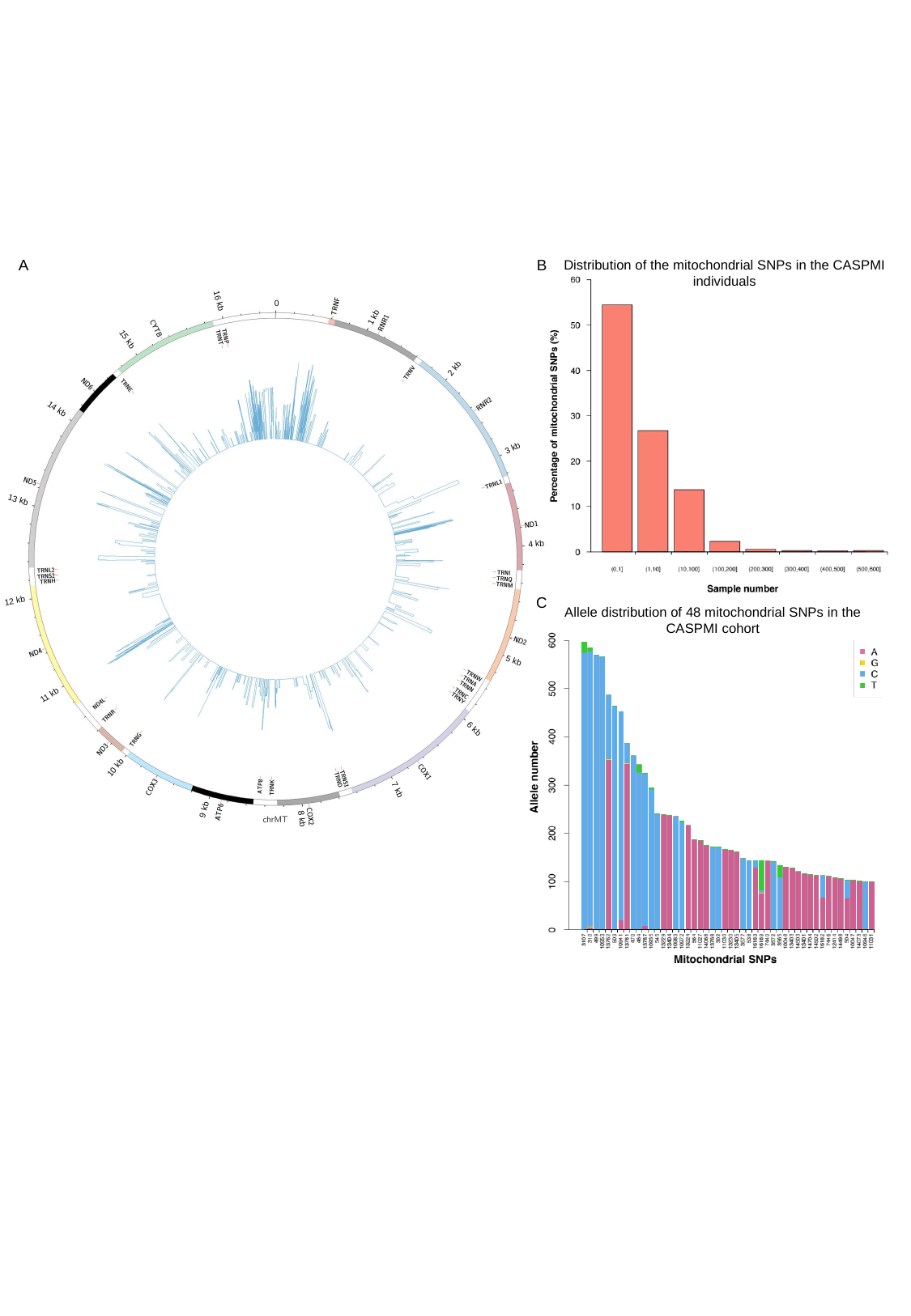

A
B
Distribution of the mitochondrial SNPs in the CASPMI individuals
16 kb
0
1 kb
15 kb
2 kb
14 kb
3 kb
13 kb
4 kb
12 kb
5 kb
11 kb
6 kb
10 kb
7 kb
9 kb
8 kb
C
Allele distribution of 48 mitochondrial SNPs in the CASPMI cohort
