# Supplementary material for: Whole Genome Analyses of Chinese Population and De Novo Assembly of A Northern Han Genome
Source: Genomics Proteomics Bioinformatics. 2019 Sep 5;17(3):229–47. doi: 10.1016/j.gpb.2019.07.002 (PMC6818495; doi:10.1016/j.gpb.2019.07.002)
Supplement: Supplementary Figure S6 — Indels identified from the CASPMI project A comparison of indels identified in the CASPMI project (pink) with those present in the dbSNP (olive green), 1KGP (gray), 1KGP EAS (green), and the 90 Han Chinese genome study (90 Han, blue) [24]. 1KGP, 1000 Genomes Project; 1KGP EAS, East Asians included in 1KGP. [file mmc6.pptx]

## Slide 1
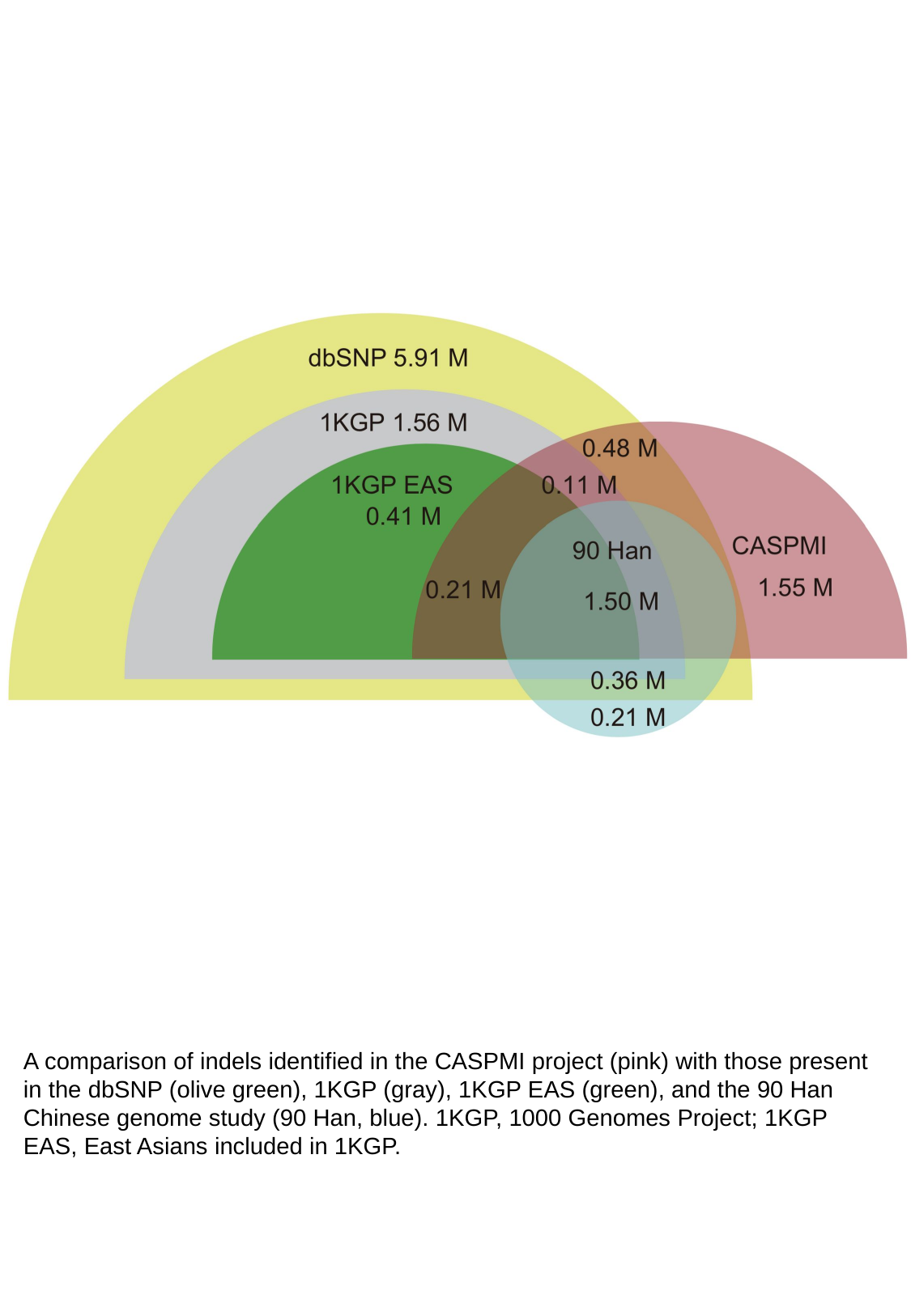

A comparison of indels identified in the CASPMI project (pink) with those present in the dbSNP (olive green), 1KGP (gray), 1KGP EAS (green), and the 90 Han Chinese genome study (90 Han, blue). 1KGP, 1000 Genomes Project; 1KGP EAS, East Asians included in 1KGP.
