# Supplementary material for: Whole Genome Analyses of Chinese Population and De Novo Assembly of A Northern Han Genome
Source: Genomics Proteomics Bioinformatics. 2019 Sep 5;17(3):229–47. doi: 10.1016/j.gpb.2019.07.002 (PMC6818495; doi:10.1016/j.gpb.2019.07.002)
Supplement: Supplementary Figure S8 — PCA plots of 1KGP EAS and the three Chinese reference genomes A. The first two principle components in PCA plot showing 1KGP EAS (including CHB, CHS, CDX, JPT, and KHV) and the three Chinese reference genomes (NH1.0, YH2.0, and HX1). B. The first two principle components in PCA plot showing 1KGP EAS with the Han Chinese populations (CHB and CHS) excluded, the three Chinese reference genomes (NH1.0, YH2.0, and HX1), as well as the northern Han and southern Han in our study (NH-CASPMI and SH-CASPMI). PCA, principal component analysis; CHB, Han Chinese in Beijing; CHS, Southern Han Chinese; CDX, Chinese Dai in Xishuangbanna; JPT, Japanese in Tokyo; KHV, Kinh in Ho Chi Minh City, Vietnam. [file mmc8.pptx]

## Slide 1
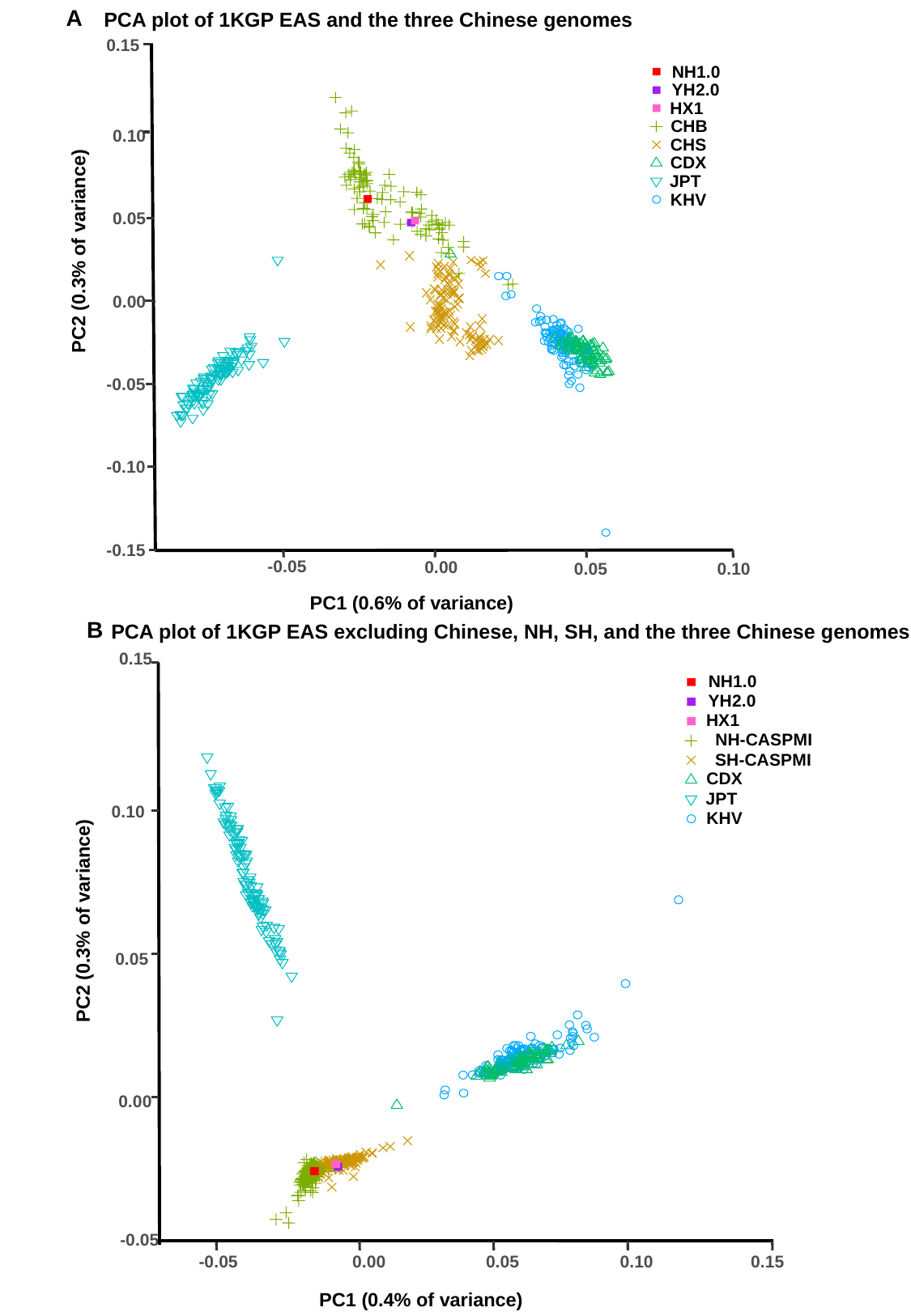

A
0.15
NH1.0
YH2.0
HX1
CHB
CHS
CDX
JPT
KHV
0.10
0.05
PC2 (0.3% of variance)
0.00
-0.05
-0.10
-0.15
-0.05
0.00
0.05
0.10
PCA plot of 1KGP EAS and the three Chinese genomes
PC1 (0.6% of variance)
B
0.15
NH1.0
YH2.0
HX1
NH-CASPMI
SH-CASPMI
CDX
JPT
KHV
0.10
PC2 (0.3% of variance)
0.05
0.00
-0.05
0.00
0.05
0.15
-0.05
0.10
PC1 (0.4% of variance)
PCA plot of 1KGP EAS excluding Chinese, NH, SH, and the three Chinese genomes
