# Supplementary material for: Whole Genome Analyses of Chinese Population and De Novo Assembly of A Northern Han Genome
Source: Genomics Proteomics Bioinformatics. 2019 Sep 5;17(3):229–47. doi: 10.1016/j.gpb.2019.07.002 (PMC6818495; doi:10.1016/j.gpb.2019.07.002)
Supplement: Supplementary Figure S13 — Allele frequency distribution of CNVs in the CASPMI cohort The y-axis shows the CNV counts with given allele frequency intervals shown on the x-axis. CNV, copy number variation. [file mmc13.docx]

## Table S13 Detailed information of the 11 hfCAS-EAS SNPs and their correlated genes

| **SNP ID** | **Ref** | **Alt** | **AF** | **EASAF** | **SASAF** | **AFRAF** | **AMRAF** | **EUR AF** | **All AF** | **Disease/trait** | **r** | **Correlated gene** | **Distance (bp)** | **Adjacent on chromosome** | **RegulomeDB score** |
| --- | --- | --- | --- | --- | --- | --- | --- | --- | --- | --- | --- | --- | --- | --- | --- |
| rs1549293 | C | T | 0.92 | 0.88 | 0.14 | 0.09 | 0.38 | 0.38 | 0.36 | Waist circumference | 0.78 | *FUS* | -54,467 | N | 2b |
| rs12627970 | A | G | 0.74 | 0.74 | 0.35 | 0.03 | 0.31 | 0.20 | 0.31 | Inflammatory bowel disease | 0.71 | *SMCR7L* | -173,175 | N | 2b |
| rs759819 | T | C | 0.81 | 0.72 | 0.29 | 0.07 | 0.36 | 0.31 | 0.33 | HDL cholesterol | 0.89 | *LILRA1* | -289,383 | N | 3a |
| rs4699934 | T | G | 0.74 | 0.75 | 0.27 | 0.42 | 0.25 | 0.13 | 0.37 | Post bronchodilator FEV1/FVC ratio | 0.79 | *PDE4D* | 38,071 | Y | 4 |
| rs4700319 | C | T | 0.74 | 0.75 | 0.27 | 0.40 | 0.25 | 0.13 | 0.37 | Post bronchodilator FEV2/FVC ratio | 0.79 | *PDE4D* | 38,020 | Y | 4 |
| rs17664743 | G | A | 0.72 | 0.65 | 0.14 | 0.31 | 0.18 | 0.22 | 0.31 | Self-reported allergy | 0.91 | *IKZF1* | -90,283 | Y | 4 |
| rs11257655 | C | T | 0.58 | 0.54 | 0.24 | 0.24 | 0.26 | 0.23 | 0.30 | Type 2 diabetes | 0.89 | *CAMK1D* | -83,086 | Y | 4 |
| rs3753242 | C | T | 0.57 | 0.60 | 0.17 | 0.02 | 0.13 | 0.06 | 0.19 | Response to antipsychotic treatment in schizophrenia (reasoning) | 0.91 | *PRKCZ* | -3421 | Y | 4 |
| rs76832595 | A | C | 0.56 | 0.54 | 0.19 | 0.04 | 0.24 | 0.13 | 0.21 | Schizophrenia | 0.83 | *KLHL29* | -28,701 | Y | 5 |
| rs1318710 | A | G | 0.73 | 0.71 | 0.31 | 0.16 | 0.26 | 0.10 | 0.30 | Food allergy | 0.74 | *EMCN* | -3039 | Y | 5 |
| rs2236496 | T | C | 0.56 | 0.56 | 0.22 | 0.11 | 0.18 | 0.19 | 0.25 | Mean corpuscular hemoglobin; mean corpuscular volume | 0.78 | *C9orf68* | 177,845 | N | 5 |

*Note*: EAS, East Asian; SAS, South Asian; AFR, African; AMR, American; EUR, European; r, Pearson correlation coefficient; N, no; Y, yes.
