# Supplementary material for: Whole Genome Analyses of Chinese Population and De Novo Assembly of A Northern Han Genome
Source: Genomics Proteomics Bioinformatics. 2019 Sep 5;17(3):229–47. doi: 10.1016/j.gpb.2019.07.002 (PMC6818495; doi:10.1016/j.gpb.2019.07.002)
Supplement: Supplementary Table S1 [file mmc16.docx]

## Table S1 Statistics of the NH1.0 whole-genome sequencing

| **Material** | **Sequencing platform** | **Library insert size** | **No. of cells/lanes** | **Sequencing data (Gb)** | **Coverage** | **Mean length** |
| --- | --- | --- | --- | --- | --- | --- |
| DNA | PacBio RSII | 10–30 kb | 132 cells | 149.8 | 49.9 × | 9.9 kb |
| DNA | 10X Genomics + Illumina HiSeqX10 | ~150 kb | 1.5 lanes | 181.2 | 60.4 × | 151 bp |
| DNA | Bionano saphyr | NA | 0.5 chip | NA | ~100 × | NA |
| DNA | Illumina HiSeq 3000 + HiSeqX10 | 300–500 bp | 2 | 237.7 | 79.2 × | 101/151 bp |
| DNA | Illumina HiSeq3000 | 3–5 kb | 1 | 20 | 6.7 × | 101 bp |
| DNA | Illumina HiSeq3000 | 5–8 kb | 1 | 22 | 7.3 × | 101 bp |
| DNA | Illumina HiSeq3000 | 8–12 kb | 1 | 27 | 8.9 × | 101 bp |

*Note*: NA, not applicable.
