# Supplementary material for: Whole Genome Analyses of Chinese Population and De Novo Assembly of A Northern Han Genome
Source: Genomics Proteomics Bioinformatics. 2019 Sep 5;17(3):229–47. doi: 10.1016/j.gpb.2019.07.002 (PMC6818495; doi:10.1016/j.gpb.2019.07.002)
Supplement: Supplementary Table S2 [file mmc17.docx]

**Table S2 Statistics of genome assembly**

|  | **10X Genomics (1)** | **PacBio (2)** | **Merged (1+2)** | **Merged + Bionano** | **10X Genomics + Bionano** | **PacBio + Bionano** |
| --- | --- | --- | --- | --- | --- | --- |
| **No. of scaffolds** | 28,438 | 6663 | 6243 | 5574 | 198 | 590 |
| **No. of gaps** | 62,874 | 0 | 6214 | 8484 | 59,983 | 14,864 |
| **Max scaffold length (bp)** | 85,445,232 | 15,335,888 | 106,371,718 | 137,497,690 | 121,009,933 | 50,285,989 |
| **Min scaffold length (bp)** | 1000 | 2946 | 1000 | 2000 | 228,641 | 166,376 |
| **Mean scaffold length (bp)** | 102,509 | 418,513 | 461,565 | 518,889 | 14,155,211 | 4,433,026 |
| **Median scaffold length (bp)** | 2065 | 89,195 | 2982 | 4082 | 4,628,451 | 2,230,440 |
| **Scaffold N50 (bp)** | 18,563,448 | 1,743,281 | 30,459,733 | 46,633,324 | 35,741,928 | 8,975,604 |
| **Scaffold N90 (bp)** | 2,691,538 | 174,414 | 4,344,446 | 6,085,355 | 8,023,970 | 2,136,959 |
| **Assembly length (bp)** | 2,915,173,181 | 2,788,557,343 | 2,881,551,743 | 2,892,287,479 | 2,802,731,936 | 2,615,485,718 |
