# Supplementary material for: Whole Genome Analyses of Chinese Population and De Novo Assembly of A Northern Han Genome
Source: Genomics Proteomics Bioinformatics. 2019 Sep 5;17(3):229–47. doi: 10.1016/j.gpb.2019.07.002 (PMC6818495; doi:10.1016/j.gpb.2019.07.002)
Supplement: Supplementary Table S3 [file mmc18.docx]

## Table S3 Sequence correction of PacBio assembly by HiSeq reads

|  | **Number of aligned reads** | |  | **Alignment rate (%)** | |  | **Mismatch rate (%)** | |  | **Indel rate (%)** | |
| --- | --- | --- | --- | --- | --- | --- | --- | --- | --- | --- | --- |
|  | **Raw** | **Corrected** |  | **Raw** | **Corrected** |  | **Raw** | **Corrected** |  | **Raw** | **Corrected** |
| Reads 1 | 390,153,391 | 390,163,215 |  | 98.422 | 98.425 |  | 0.575 | 0.571 |  | 0.123 | 0.023 |
| Reads 2 | 388,982,133 | 389,008,303 |  | 98.127 | 98.134 |  | 0.835 | 0.834 |  | 0.121 | 0.023 |
| PAIR | 779,135,524 | 779,171,518 |  | 98.275 | 98.279 |  | 0.704 | 0.702 |  | 0.122 | 0.023 |
