# Supplementary material for: Whole Genome Analyses of Chinese Population and De Novo Assembly of A Northern Han Genome
Source: Genomics Proteomics Bioinformatics. 2019 Sep 5;17(3):229–47. doi: 10.1016/j.gpb.2019.07.002 (PMC6818495; doi:10.1016/j.gpb.2019.07.002)
Supplement: Supplementary Table S4 [file mmc19.docx]

**Table S4 Comparison of mapping profiles between GRCh38 and NH1.0**

| **Population** | **Ethnicity** | **Sample ID** | **Number of filtered reads** | **GRCh38** | | |  | **NH1.0** | | |
| --- | --- | --- | --- | --- | --- | --- | --- | --- | --- | --- |
|  |  |  |  | **Number of mapped reads** | **Mapping rate (%)** | **Mismatch rate (%)** |  | **Number of mapped reads** | **Mapping rate (%)** | **Mismatch rate (%)** |
| SAS | Punjabi | HG01583 | 235,364,682 | 235,105,058 | 99.89 | 0.41 |  | 233,605,717 | 99.25 | 0.47 |
| (South Asian) | Bengali | HG03832 | 284,713,570 | 283,505,666 | 99.58 | 0.49 |  | 282,017,140 | 99.05 | 0.53 |
|  | Indian | HG03779 | 388,327,082 | 387,233,976 | 99.72 | 0.58 |  | 385,551,655 | 99.29 | 0.58 |
| AFR (African) | African-Caribbean | HG01985 | 332,485,594 | 331,567,023 | 99.72 | 0.47 |  | 330,184,602 | 99.31 | 0.49 |
|  | Esan | HG03367 | 337,223,538 | 336,501,877 | 99.79 | 0.51 |  | 334,658,440 | 99.24 | 0.56 |
|  | Mende | HG03060 | 387,894,734 | 386,423,401 | 99.62 | 0.45 |  | 384,257,740 | 99.06 | 0.50 |
| EUR | British | HG00154 | 283,372,146 | 282,533,956 | 99.70 | 0.47 |  | 281,460,026 | 99.33 | 0.50 |
| (European) | Finnish | HG00268 | 560,101,088 | 557,167,919 | 99.48 | 0.58 |  | 555,341,687 | 99.15 | 0.58 |
|  | Spanish | HG01518 | 283,627,476 | 281,612,784 | 99.29 | 0.39 |  | 280,269,792 | 98.82 | 0.44 |
| AMR | Colombian | HG01374 | 229,753,612 | 229,138,704 | 99.73 | 0.40 |  | 228,235,577 | 99.34 | 0.41 |
| (American) | Puerto Rican | HG00553 | 199,330,424 | 198,158,920 | 99.41 | 0.47 |  | 197,118,250 | 98.90 | 0.53 |
|  | Peruvian | HG01893 | 351,555,306 | 350,757,175 | 99.77 | 0.49 |  | 349,391,112 | 99.38 | 0.48 |
| CHB | Han Chinese | ERR234331 | 235,343,638 | 234,696,718 | 99.73 | 0.54 |  | 233,963,680 | 99.41 | 0.49 |
| (Chinese in Beijing) | Han Chinese | ERR234333 | 214,007,348 | 213,329,278 | 99.68 | 0.54 |  | 212,705,981 | 99.39 | 0.50 |
|  | Han Chinese | SRR189816 | 248,849,782 | 247,873,321 | 99.61 | 0.92 |  | 247,128,961 | 99.31 | 0.88 |
