# Supplementary material for: Whole Genome Analyses of Chinese Population and De Novo Assembly of A Northern Han Genome
Source: Genomics Proteomics Bioinformatics. 2019 Sep 5;17(3):229–47. doi: 10.1016/j.gpb.2019.07.002 (PMC6818495; doi:10.1016/j.gpb.2019.07.002)
Supplement: Supplementary Table S5 [file mmc20.docx]

Table S5 SV comparison of the three Chinese reference genomes

| **SV types** | **Number of SVs** | **Number of repetitive regions (%)** | **Number of exonic regions (%)** | **Number of intronic regions (%)** | **Number of intragenic regions (%)** | **Number of intergenic regions (%)** | **Number of shared SVs (%)** |
| --- | --- | --- | --- | --- | --- | --- | --- |
| **Deletions** |  |  |  |  |  |  |  |
| **YH2.0** | 6402 | 3679 (57.5%) | 117 (1.8%) | 3180 (49.7%) | 3239 (50.6%) | 2546 (39.8%) | 1497 (23.4%) |
| **HX1** | 6736 | 3705 (55.0%) | 111 (1.6%) | 3140 (46.6%) | 3198 (47.5%) | 2849 (42.3%) | 1820 (27.0%) |
| **NH1** | 7252 | 3859 (53.2%) | 162 (2.2%) | 3475 (47.9%) | 3565 (49.2%) | 3025 (41.7%) | 1901 (26.2%) |
| **Insertions** |  |  |  |  |  |  |  |
| **YH2.0** | 6514 | 4221 (64.8%) | 230 (3.5%) | 3545 (54.4%) | 3651 (56.0%) | 2775 (42.6%) | 872 (13.4%) |
| **HX1** | 10,056 | 6667 (66.3%) | 355 (3.5%) | 5429 (54.0%) | 5596 (55.6%) | 4411 (43.9%) | 1149 (11.4%) |
| **NH1** | 11,361 | 7565 (66.6%) | 374 (3.3%) | 6337 (55.8%) | 6510 (57.3%) | 4793 (42.2%) | 1341 (11.8%) |
