# Supplementary material for: Whole Genome Analyses of Chinese Population and De Novo Assembly of A Northern Han Genome
Source: Genomics Proteomics Bioinformatics. 2019 Sep 5;17(3):229–47. doi: 10.1016/j.gpb.2019.07.002 (PMC6818495; doi:10.1016/j.gpb.2019.07.002)
Supplement: Supplementary Table S6 [file mmc21.docx]

**Table S6 Stratification and application of CASPMI samples**

| **Category** | **Number** | **Subtotal** | **Total** | **Application** |
| --- | --- | --- | --- | --- |
| Male | 246 |  | 597 | Association analysis |
| Female | 351 |  |  |  |
| Northern Han | 339 | 455 | 597 | Phenotype association study, population differentiation |
| Southern Han | 116 |  |  |  |
| Mixed ancestry | 73 |  |  |  |
| Other ethnic groups | 42 |  |  |  |
| NA* | 27 |  |  |  |

*Note*: *, data are not applicable for analysis due to missing in ancestry or ethnic report, or failure for kinship test.
