# Supplementary material for: Whole Genome Analyses of Chinese Population and De Novo Assembly of A Northern Han Genome
Source: Genomics Proteomics Bioinformatics. 2019 Sep 5;17(3):229–47. doi: 10.1016/j.gpb.2019.07.002 (PMC6818495; doi:10.1016/j.gpb.2019.07.002)
Supplement: Supplementary Table S7 [file mmc22.docx]

**Table S7 SNP distribution in mitochondrial genes**

| **Gene ID** | **SNP number** | **Gene ID** | **SNP number** |
| --- | --- | --- | --- |
| *ND5* | 142 | *TRNE* | 6 |
| *CYTB* | 85 | *TRNI* | 5 |
| *ND4* | 84 | *TRNV* | 4 |
| *COX1* | 75 | *TRNW* | 3 |
| *ND1* | 66 | *TRND* | 3 |
| *RNR2* | 55 | *TRNH* | 3 |
| *ATP6* | 53 | *TRNN* | 3 |
| *ND2* | 48 | *TRNC* | 3 |
| *COX3* | 48 | *TRNS1* | 3 |
| *RNR1* | 44 | *TRNK* | 3 |
| *ND6* | 31 | *TRNM* | 3 |
| *COX2* | 28 | *TRNL1* | 3 |
| *ND3* | 22 | *TRNA* | 2 |
| *ATP8* | 20 | *TRNR* | 2 |
| *TRNP* | 10 | *TRNS2* | 2 |
| *TRNG* | 9 | *TRNL2* | 1 |
| *TRNT* | 9 | *TRNQ* | 1 |
| *ND4L* | 9 | *TRNY* | 1 |
| *TRNF* | 8 |  |  |

## 
