# Supplementary material for: Whole Genome Analyses of Chinese Population and De Novo Assembly of A Northern Han Genome
Source: Genomics Proteomics Bioinformatics. 2019 Sep 5;17(3):229–47. doi: 10.1016/j.gpb.2019.07.002 (PMC6818495; doi:10.1016/j.gpb.2019.07.002)
Supplement: Supplementary Table S10 [file mmc25.docx]

## Table S10 Age distribution in the NH and SH males of the CASPMI cohort

| **Age** | **Number of males** | **Number of NH males** | **Number of SH males** |
| --- | --- | --- | --- |
| 20**–**29.9 | 14 | 9 | 5 |
| 30**–**34.9 | 69 | 52 | 17 |
| 35**–**39.9 | 52 | 37 | 15 |
| 40**–**44.9 | 25 | 17 | 8 |
| ≥ 45 | 40 | 30 | 10 |
| Total | 200 | 145 | 55 |
