# Supplementary material for: Whole Genome Analyses of Chinese Population and De Novo Assembly of A Northern Han Genome
Source: Genomics Proteomics Bioinformatics. 2019 Sep 5;17(3):229–47. doi: 10.1016/j.gpb.2019.07.002 (PMC6818495; doi:10.1016/j.gpb.2019.07.002)
Supplement: Supplementary Table S11 [file mmc26.docx]

## Table S11 Waist circumferences and genotypes of rs1549293 in the CASPMI cohort

| **Gender** | **Genotype** | **No. of participants** | |  | **Average circumference (cm)** | | ***P* value** |
| --- | --- | --- | --- | --- | --- | --- | --- |
|  |  | **NH** | **SH** |  | **NH** | **SH** |  |
| Male | TT | 122 | 41 |  | 87.6 | 82 | 8.81E–05 |
|  | CT | 22 | 13 |  | 84.2 | 82.6 | 0.2529 |
| Female | TT | 174 | 50 |  | 73.8 | 72.5 | 0.1369 |
|  | CT | 19 | 10 |  | 73.5 | 74 | 0.4608 |
