# Supplementary material for: Whole Genome Analyses of Chinese Population and De Novo Assembly of A Northern Han Genome
Source: Genomics Proteomics Bioinformatics. 2019 Sep 5;17(3):229–47. doi: 10.1016/j.gpb.2019.07.002 (PMC6818495; doi:10.1016/j.gpb.2019.07.002)
Supplement: Supplementary Table S16 [file mmc31.docx]

## Table S16 Comparison of SVs identified in CASPMI with those present in dbVar and DGV

| **SV type** | **No. of SVs** | **Compared with dbVar** | |  | **Compared with DGV** | |  | **Compared with dbVar & DGV** | |
| --- | --- | --- | --- | --- | --- | --- | --- | --- | --- |
|  |  | **No. of common SVs** | **No. of unique SVs** |  | **No. of common SVs** | **No. of unique SVs** |  | **No. of common SVs** | **No. of unique SVs** |
| **Insertion** | 2249 | 1530 | 719 |  | 682 | 1567 |  | 1603 | 646 |
| **Deletion** | 102,663 | 3335 | 99,328 |  | 37,403 | 65,260 |  | 37,593 | 65,070 |
| **Inversion** | 38 | 24 | 14 |  | 22 | 16 |  | 26 | 12 |
| **CNV** | 1432 | 1403 | 29 |  | 1417 | 15 |  | 1428 | 4 |
