# Supplementary material for: Whole Genome Analyses of Chinese Population and De Novo Assembly of A Northern Han Genome
Source: Genomics Proteomics Bioinformatics. 2019 Sep 5;17(3):229–47. doi: 10.1016/j.gpb.2019.07.002 (PMC6818495; doi:10.1016/j.gpb.2019.07.002)
Supplement: Supplementary Table S17 [file mmc32.docx]

## Table S17 Annotation of repetitive elements overlapping with SNVs, indels, and three types of SVs in the CASPMI cohort

| **Category** | | | **SNVs Number** | **Indels** | |  | **Deletions** | |  | **Insertions** | |  | **Inversions** | |
| --- | --- | --- | --- | --- | --- | --- | --- | --- | --- | --- | --- | --- | --- | --- |
|  |  |  |  | **Number** | **Mean length (bp)** |  | **Number** | **Mean length (bp)** |  | **Number** | **Mean length (bp)** |  | **Number** | **Mean length (bp)** |
| Total variations | | | 24,850,606 | 3,851,223 |  |  | 102,664 |  |  | 2250 |  |  | 39 |  |
| Variations overlapping with repeat regions | Mobile element | SINE | 3,486,614 | 786,879 | 2.23 |  | 20,830 | 95.9 |  | 119 | 181.39 |  | 0 | NA |
|  |  | LINE | 4,956,412 | 626,133 | 1.93 |  | 10,595 | 110.18 |  | 252 | 180.03 |  | 0 | NA |
|  |  | LTR | 2,410,531 | 205,310 | 2.18 |  | 3666 | 102.11 |  | 122 | 129 |  | 3 | 376.67 |
|  |  | DNA elements | 880,839 | 95,457 | 2.35 |  | 1188 | 83.23 |  | 49 | 136.88 |  | 1 | 84 |
|  |  | Total^$^ | 11,734,396 | 1,713,779 |  |  | 36,279 |  |  | 542 |  |  | 4 |  |
|  | Tandem repeat | RNA | 11,615 | 1205 | 2.55 |  | 64 | 98.83 |  | 0 | NA |  | 0 | NA |
|  |  | Satellites | 91,384 | 12,321 | 1.84 |  | 1076 | 668.26 |  | 32 | 1858.97 |  | 0 | NA |
|  |  | Simple repeats | 156,904 | 557,070 | 3.4 |  | 7079 | 88.56 |  | 254 | 123.98 |  | 8 | 113.63 |
|  |  | Low complexity | 121,824 | 116,986 | 2.85 |  | 1593 | 86.29 |  | 92 | 106.33 |  | 0 | NA |
|  |  | Total^&^ | 381,727 | 687,582 |  |  | 9812 |  |  | 378 |  |  | 8 |  |
|  | Complex |  | 8103 | 34,264 | 4.44 |  | 24,990 | 754.93 |  | 808 | 7354.61 |  | 13 | 3460.62 |
|  | Other |  | 12,763 | 9412 | 2.4 |  | 1255 | 158.99 |  | 12 | 125.25 |  | 0 | NA |
|  | Unknown |  | 11,097 | 882 | 2.04 |  | 11 | 80.36 |  | 6 | 87.5 |  | 0 | NA |
|  | Total^#^ | | 12,148,086 (48.88%) | 2,445,919 (63.51%) |  |  | 72,347 (70.47%) |  |  | 1746 (77.60%) |  |  | 25 (64.10%) |  |

*Note*: Complex refers to the variants that were simultaneously annotated by more than 2 types of repeat classes. SINE, short interspersed nuclear elements; LINE, long interspersed nuclear element; LTR, long tandem repeat; RNA, tandem repeat of rRNA or tRNA. Total variations in mobile elements, tandem repeats, and all repeat regions are indicated with $, &, and # in superscripts, respectively. Percentage of variations overlapping with repeat regions among the total variations is shown in parenthesis.
